# The “Selfish Cell”: The Central Role of Metabolism in Relaying Environmental Stimuli and Controlling Cellular Function

**DOI:** 10.3390/biology15191790

**Published:** 2026-10-08

**Authors:** Sabina Di Matteo, Davide Gnocchi

**Affiliations:** 1 Departmental Faculty of Medicine, UniCamillus- Saint Camillus International University of Health and Medical Sciences, 00131 Rome, Italy; sabina.dimatteo@unicamillus.org; 2 IRCCS San Camillo Hospital, 30126 Venice, Italy

**Keywords:** selfish cell, metabolism, epigenetics, chronic non-communicable diseases (CNCDs), tumors, translational applications

## Abstract

Science is dynamic, and novel models to better understand experimental observations are very important for its advancement. In the context of cell biology, knowledge about how cells behave in response to extracellular stimuli, and how these processes are dysregulated in pathologic conditions, is one of the challenges of biological research nowadays. From a theoretical point of view, the discovery of the double-helix model of DNA structure led to the idea that most aspects of a cell’s behavior could be understood by knowing the sequence and the rules regulating the expression of genes. A paradigm of this way of thinking is the theory of the “selfish gene” by Richard Dawkins. Indeed, this approach is being found to have several limitations, especially in the interpretations of complex pathologies, such as cancer, metabolic or neurodegenerative diseases. For this reason, a paradigm shift should be considered to provide a better modeling of the biological processes that control cellular behavior and physiology. In particular, a central role should be given to metabolism as a relay between the environment and microenvironment and cell function, by driving epigenetic modifications. For these reasons, a new “selfish cell” model should be introduced, which could prove useful to better explain complex diseases and find more effective translational approaches.

## Simple Summary

The use of correct models is important in science, because they provide the proper theoretical toolkit to understand the process under investigation. In biological sciences, important examples are Darwin’s theory of evolution or Lynn Margulis’ endosymbiotic theory of the origin and function of mitochondria. For what concerns cell biology, a line of theoretical division has been drawn after the discovery of the double-helix model of DNA structure. After that, most efforts have been made to increase the knowledge in this field, which we now know as molecular biology or molecular genetics. Still, this special attention to understanding the molecular details of cellular biology had the consequence of losing the focus on a more systemic and broad-range spectrum of investigation. The idea that most about a cell’s behavior could be interpreted by knowing sequence and transcriptional rules of genes provided the background for the formulation of Dawkins’ theory of “selfish gene”. However, this “reductionist” and mechanistic approach may prove not adequate to properly describe the complexity which per se defines a eukaryotic, metazoan cell. Thus, a paradigm shift should be introduced in our way of approaching the study and the understanding of a cell’s behavior: from the “selfish gene” to a “selfish cell” theoretical framework.

## 1. Introduction

The use of adequate models is very important in science because, with this choice, the correct interpretation of experimental data is drawn. Several examples can be mentioned in this context. In biology, Darwin’s evolution theory is one of the most known and popular, and still at the present time provides a basis for the understanding not only of evolutive processes, but also, sensu lato, of the behavior of cells. Indeed, in recent years, a “Neo-Darwinian” approach has been raised, which claims that Darwin’s ideas can be extended to the study of cellular processes, putting genes at the center of a cell’s behavior. This approach follows a big breakthrough in the past century’s biochemical science, namely the discovery of the double helix structure of DNA by James Watson, Francis Crick and Rosalind Franklin. This led to the conception of the so-called “central dogma” of molecular biology, which can be summarized as follows: DNA → RNA → Proteins, with just a few rearrangements that have been added to include, for example, retrotranscription or regulation by microRNA (miRNA), or exceptions like prion proteins. Thus, the discovery of the double-helix model of DNA set the stage for the central dogma, and this provided a theoretical background for the “Neo-Darwinism” and, consequently, for the “selfish gene” theory by Richard Dawkins. According to this view, in the genes there is all of the information needed to make a cell work properly and to build a whole organism [1,2,3]. The idea is that knowing the sequence of genes is enough and sufficient to fully understand everything about the life processes of biological organisms, humans included [4]. This model is of course tempting, as linear and straightforward perspectives always are. Probably such a mental scheme has also been strengthened by a “computer way of thinking”, which became popular in the same years. Still, this approach is probably too “reductionist” to correctly modelise and explain the complexity of cell biology, and, in general, inadequate to describe physiological processes [5]. Indeed, Denis Noble wrote that, today, neither Darwinian process would be a “Neo-Darwinian” [2]. The problem here is that complex systems require complex tools to be fully and properly understood, and just knowing gene sequences and the protein products of the genes is not enough [6]. In fact, the “Human Genome Project” did not hold the translational promises for what concerns complex diseases, like neurodegenerative and metabolic disorders, and tumors and was informative mainly for phylogenetic purposes [5]. Indeed, the weakness of the outcomes of the “Human Genome Project” for translational purposes has been underlined by authoritative experts [7]. In particular, the applicability of DNA testing is limited from a diagnostic point of view, even after the application of “polygenic risk scores”, which are probabilistic and have limited precision [8]. Finally, regarding pharmacogenomics, despite benefits in avoiding rare serious drug reactions, clinical trials did not provide evidence of utility of the pharmacogenomic approach for many common classes of diseases [9,10], including cancer [11,12]. In this perspective, the idea of personalized and precision medicine, though theoretically useful, has been overestimated [7]. The reason is the misconception that gene variants can improve the understanding of disease biology, and thus be translated into pharmacological therapy. The assumption here is that the genome is the *primum movens* of every cellular event. Still, the stimuli for gene action are environmental; hence, the environment is the main cause, not the gene itself [7]. Hence, a paradigm shift is necessary to fully understand the complexity of biological systems [4]. This will also help in improving therapeutic approaches and disease management.

As underlined by Denis Noble: “Gene-centrism has had influence way beyond biology or clinical medicine. The peak influence in the medical sciences was the representation of the genome as the “book of life” and “Instead through genome-wide sequencing we have encountered a failure of gene-centrism. Most association levels are small, so much so that we can be sure that such methods do not reveal causation. In reality, the regulatory networks simply buffer organisms from most genetic changes”. And also: “…differences [i.e., association scores] cannot reveal the totality of functions that a gene may be involved in, since they cannot reveal all the effects that are common to the wild and mutated types. We may be looking at the tip of an iceberg. And we may be looking at the wrong tip since we may be identifying a gene through the pathological effects of just one of its mutations rather than by what it does for which it must have been selected. This must be true of so-called oncogenes, since causing cancer his unlikely to be function for which the genes were selected… Identifying genes by differences in phenotype correlated with those in genotype is therefore hazardous. Many, probably most, genetic modifications are buffered” [3,4]. In cancer, for example, most of the drugs currently employed in clinical treatments did not manage to bring substantial improvement to the treatment of cancer patients. The best results obtained along this way are limited to a few months’ extension of the life expectancy of patients, with an average of around four months. Moreover, most of these therapies led to relapses, which, in most of the cases, comes back with higher malignancy than the primary tumor [13]. As underlined in this quote by Robert Weinberg: “the most potent carcinogens are actually not mutagens” [14].

For these reasons, it is important to start broadening the theoretical toolkit that we use when we approach the study of cell biology, physiology and pathology, and introduce a “paradigm shift” from the “gene-centric”, “selfish gene” perspective to a “cellular-centric, “selfish cell” model. This novel perspective will help in better understanding the physiology of cells and not just the molecular processes that lie at a downstream level in the intricate and complex machinery that rules cell behavior. Such molecular processes are just a consequence or a response to environmental and, in turn, microenvironmental stimuli. In general, we can talk about the “exposome”, referring to all of the stimuli or challenges we face as human beings [15]. Hence, the scientific question could be: How do our organisms face pollution, low-quality nutrition, poor quality of sleep and psychosocial stress? Also, how does this “exposome” affect our metabolism, cognition and the onset of CNCDs? Moreover, how important is the role of metabolism in translating “exposome” or “envirome” challenges in pathological settings? Finally, how are all these challenges interconnected, and can some emergent effects and/or functions arise? In this context, the “selfish cell” model can provide a more adequate theoretical tool to answer these questions in the context of complex biological systems as we are. So, here, we will give our contribution by providing a framework to better structure these concepts. In a way, Dawkins himself accepted 10 years ago that a change in perspective is necessary, as evidenced in this quote: “So many exciting things are fast happening in the world of genomics, it would seem almost inevitable—even tantalising—that a book with the word ‘gene’ in the title would, forty years on, need drastic revision if not outright discarding” (Dawkins, 2016, *The Selfish Gene: 40th Anniversary edition*).

**Figure 1:**
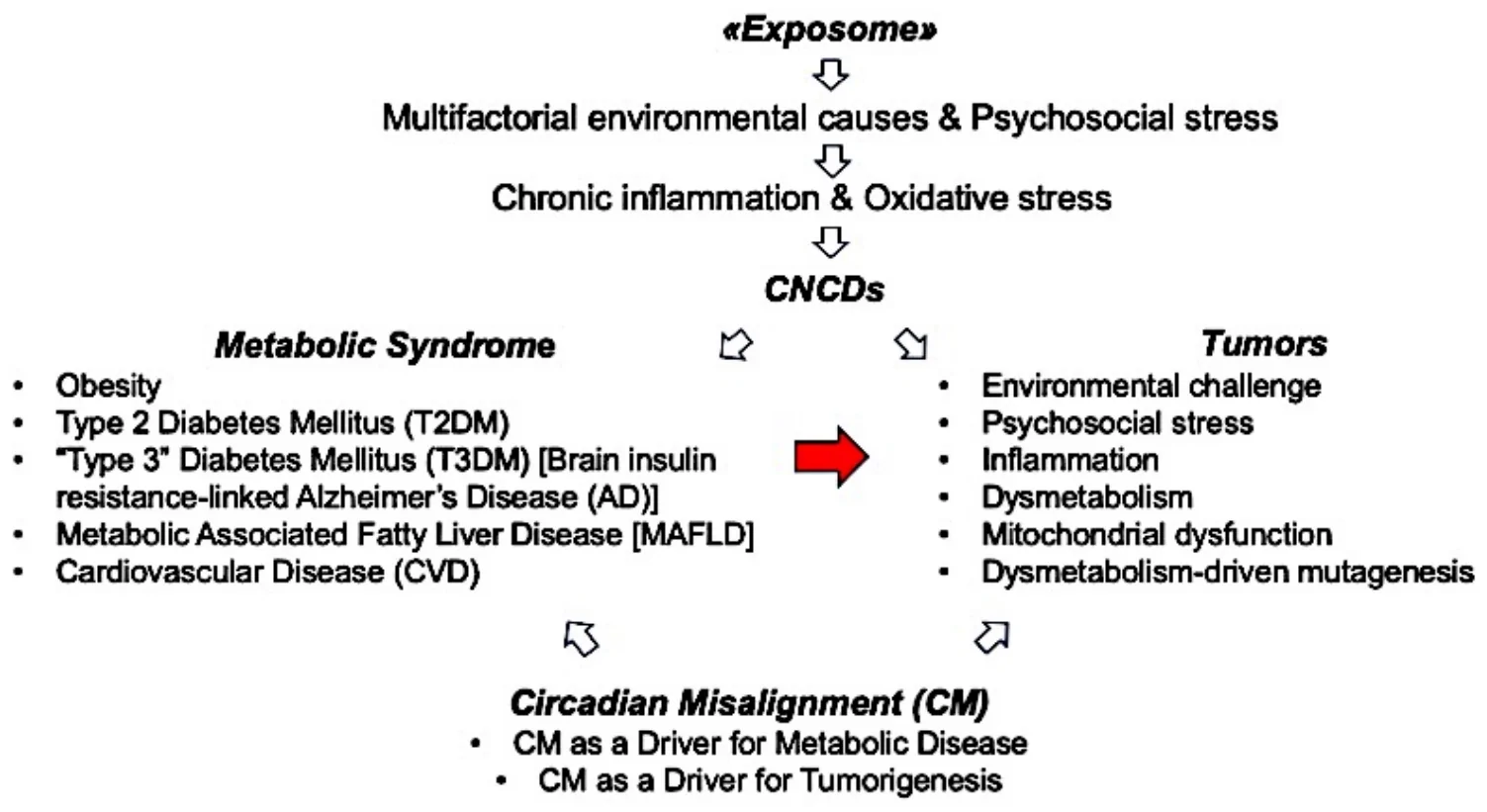
Graphical summary of the conceptual framework followed in this review.

Figure 1 summarizes the topics we reviewed.

To write this review, we employed a MEDLINE search (via PubMed) for articles published from database inception up to July 2026, with no language restrictions, using the following search terms: “Chronic non-communicable diseases, circadian misalignment, human genome project, Warburg effect, tumor metabolism, tumor immunotherapy, lactate, cancer epigenetics”.

## 2. “Selfish Cell” vs. “Selfish Gene”

In the purpose of understanding how a cell’s behavior is regulated and which mechanisms are involved, a good standpoint is to discuss the basis of the so-called “selfish gene” model proposed by Richard Dawkins almost half a century ago. As said above, this model has its roots in the discovery of the DNA double helix structure. After this achievement, and the formulation of the central dogma of molecular biology, many scientists started to think from a molecular biology perspective, and to believe that most of the knowledge needed to understand organisms could be found in DNA.

One of the implications and claims of this approach is that knowing the sequence and product of genes could provide all the information needed to build an organism and to draw a complete functional map. Indeed, this approach has several flaws, which are more evident when the time comes to apply it in translational applications [5,6].

For this reason, here, we propose that, to better manage organisms’ complexity, a more useful working model would be that of a “selfish cell”, a biological concept able to integrate complex environmental and microenvironmental stimuli, and, in turn, to orchestrate complex biological responses. From a translational point of view, a “selfish cell” model could provide a more adequate theoretical tool. Here, we will describe and summarize the many convincing pieces of evidence that support the scientific and theoretical framework of a “selfish cell”.

## 3. Metabolism as a Relay Between Environment and Cell Function

For the purpose of better understanding the behavior of a cell’s function, a deeper knowledge of the role of metabolism is of key relevance. Metabolism is, by definition, the inclusion of anabolic and catabolic reactions, so the sum of biosynthesis/anaplerosis and catabolism/energy producing processes. The central role of metabolism in the regulation of cell function and homeostasis can be found in many processes and is independent of genetic regulation. Indeed, changes in metabolic function have a wide spectrum of outcomes, which encompasses the etiology of many diseases. In this context, mitochondria have a very important role as a hub of metabolic regulation.

### Metabolic Basis of Chronic Non-Communicable Diseases (CNCDs)

Chronic non-communicable diseases (CNCDs) are showing an alarming upward trend in the last three decades [16]. Despite the efforts to find a clear genetic basis for these diseases, so far, no solid progress has been made. The reason is that CNCDs have their roots in the complex interaction between environmental triggers and cellular response. In general, environmental stimuli are the main drivers of CNCDs, because they interact with cellular systems in multiple ways. In addition, it is very important to include in the category of environmental stimuli also the so-called psychosocial stress, which is able to trigger sustained modifications to our internal milieu, promoting the onset of many CNCDs. Psychosocial stress can be defined as the result of the many adverse behavioral and social interactions we experience in our everyday life.

Table 1 summarizes examples of CNCDs with their epidemiological burden of associated deaths. The main metabolic root of these diseases is also reported.

Troubles, internal conflicts, interpersonal conflicts, anxious states, chronic stress, for example, are translated by our body in several ways, typically by increasing cortisol and adrenal hormones [17,18,19]. In addition, as we will see in the next paragraphs, psychosocial and chronic stress can strongly dysregulate our internal clock, namely the circadian clock machinery. This circadian misalignment has been shown to predispose to metabolic diseases and tumorigenesis [20]. Thus, a modification of cell function without direct involvement of genetic mutations or changes. We will better describe these subjects in the next paragraphs of this review.

Metabolism is therefore central, because the metabolic machinery holds the ability to sense microenvironmental changes and to translate them into functional changes. This implies an important role of epigenetic modifications, which involve both histone and non-histone proteins, as we will see in more detail in the next paragraphs.

## 4. Tumor as a Metabolic Disease

### 4.1. Tumor as a Complex Disease

Tumors are, by definition, complex systems; thus, as complex systems, they should be studied and interpreted. The effort for a correct interpretation of tumorigenesis is huge, of course. The traditional theory, on which most of the therapeutic approaches are based, is the so-called somatic mutations theory (SMT). According to this theory, cancer arises when in the normal cell a “threshold” of somatic mutations is reached in some “key” genes, controlling cell growth and proliferation. This theory implies a high degree of aleatoric events, and for this reason it was suggested to Tomasetti and Vogelstein as what they called the “bad luck theory”, published in *Science* in 2015 [21]. According to this view, random mutations, in a way, “bad luck”, are “the major contributors to cancer overall, often more important than either hereditary or external environmental factors.” This view raised many reactions, including that of International Agency for Cancer Research (IARC), which in a press release a few days after the publication of the paper stated: “If misinterpreted, this position could have serious negative consequences from both cancer research and public health perspectives.” Also: “Concluding that ‘bad luck’ is the major cause of cancer would be misleading and may detract from efforts to identify the causes of the disease and effectively prevent it” (IARC—Press Release N° 231—13 January 2015). This IARC statement has been corroborated and strengthened in 2016 by Wu and colleagues, who found that the contribution of intrinsic risk factors to tumorigenesis is very limited, concluding that “cancer risk is heavily influenced by extrinsic factors” [22]. A follow-up paper published in *Science* in 2017 recognized the importance of environmental factors in tumorigenesis [23]. However, it is important to underline here that despite the recognition of the presence of mutations in cancers and their generation by DNA replication errors, this does not mean that mutations are the first cause of tumorigenesis. Indeed, a high mutational rate can be induced in cells undergoing a fermentative metabolic rewiring [24]. We will examine these concepts with further details later in this review.

In general, the SMT also implies a complex “selection” process, different for each tumor type, in order to explain the high degree of heterogeneity observed in cancers. Overall, according to this theory, somatic mutations are the main driver of cancerogenesis. For this reason, most of the currently approved therapeutic and pharmacological approaches are based on SMT. Examples are tyrosine kinase inhibitors (TKIs) targeting mutated sites on growth factor receptors, or monoclonal antibodies (mAbs) directed to specific targets. Types of mAbs include: trastuzumab, which blocks EGFR2 (HER2), bevacizumab, which blocks Vascular Endothelial Growth Factor (VEGF), and atezolizumab, which targets Programmed Death-Ligand 1 (PD-L1). Of note, many of the approaches based on SMT proved to be not effective in the long range and hence not curative, and with relapse after a few months [25]. In addition, a plethora of side effects have been described for this class of drugs, such as fever, gastrointestinal problems (nausea, vomiting, colitis, diarrhea), musculoskeletal pain, tiredness, hypertension, proteinuria, and cutaneous eruptions [26]. Several serious side effects have also been described in the long term [27]. Moreover, it has been recently found and published in a *Nature* paper that children after different types of chemotherapy had a significantly higher presence of somatic mutations. The authors concluded: “These data highlight opportunities for treatment de-escalation” [28]. Interestingly, as we will describe in more detail in the next paragraphs, it has been recently proposed that ancient and evolutionarily conserved adaptive mutation responses typical of bacteria can be similarly found in cancer and be the source of genomic instability. Biological adaptation through Stress-Induced Mutagenesis (SIM) might be the basis of the capacity of tumors to adapt in the face of harsh selective pressures such as treatments and indicates that the current “hit it hard” approaches to cancer therapy could be counterproductive [29].

Just a few exceptions can be mentioned for what concerns liquid tumors, due to their lower biological complexity, resilience and variability compared to solid tumors. Moreover, they are metabolically “weaker”, since they have a lower Spare Respiratory Capacity (SRC), as demonstrated in a *Blood* paper in 2015 on Acute Myeloid Leukemia (AML) [30]. This characteristic makes leukemias and, in general, liquid tumors more susceptible to oxidative stress, and easier to kill. Therefore, the effectiveness of some of the current pharmacological approaches in liquid tumors may be viewed as a particular case, or an exception that confirms a more general picture.

It is worth mentioning here that much counterevidence has been found against SMT, such as: (i) not all tumors are correlated with specific mutations; (ii) tumors can develop even in the absence of specific mutated genes; and (iii) some tumors do not present mutations at all [13,31].

During the last few decades, new ideas started to emerge in the attempt to provide innovative perspectives and possible interpretative concepts to better understand the onset and development of tumors. One of these theories was proposed in 1999 by Carlos Sonnenschein and Ana Soto and is defined as Tissue Organization Field Theory (TOFT). According to this view, tumorigenesis is an issue related to tissue homeostasis, rather than a single cell transformation due to somatic mutations. In particular, TOFT postulates that dangerous substances exert a destabilizing action at the tissue level, triggering a loss of homeostasis at the tissue. This theory postulates the tissue as a form of collaboration between cells, which can be lost whenever the cellular microenvironment becomes “hostile”, due to specific stimuli, such as, for example, asbestos fibers or endocrine disruptors. So, what is disrupted is the “tissue/morphogenetic field”, which means the sum of the structural and biochemical interactions that keep the tissue as it is, defining its nature. Another pillar of this theory is that the cell’s default state is not quiescence, but proliferation: this condition is kept tamed in normal healthy conditions. This homeostatic condition is maintained until perturbing stimuli unleash the proliferative “true” nature of cells, which is uncontrolled proliferation. For many reasons, TOFT offers interesting answers and perspectives to better explain tumorigenesis, if compared to SMT. For example, it highlights the importance of the cellular microenvironment and extracellular matrix, together with the role of fibroblasts. TOFT is also in line with the morphological changes observed in the organs when a tumor arises, meaning the loss of cellular shape and the onset of dedifferentiation [32,33,34,35]. Table 2 provide a synoptic summary of the main charateristics of SMT ad TOFT.

Another two interesting theories about the origin of tumorigenesis are the so-called “atavistic” theory and the “metabolic/mitochondrial” theory. These two theories have some points in common, as we will see hereafter. The “atavistic” theory of tumorigenesis postulates that cancer arises as a consequence of a process of awakening of cellular processes typical of unicellular eukaryotes, with particular reference to those of Proterozoic age [36,37,38,39]. Indeed, a *Nature* paper published in 2004 proved that in our genomes there are still those biochemical modules which allowed unicellular eukaryotes of Proterozoic age to survive the very challenging conditions of that era [40]. Very recently, another *Nature* paper corroborated this view [41]. So, this challenging environment was characterized by high acidity, low oxygen (O_2_) and elevated sulfhydryl/sulfidic stress (euxinic environment) [42,43]. In this perspective, the “atavistic” theory of tumorigenesis is in line with the theory of cancer as a loss of multicellular cooperation proposed by Athena Aktipis and colleagues [44]. In the last 10 years, four seminal papers by Anna Trigos from the Peter MacCallum Cancer Centre in Melbourne showed, with a bioinformatic approach, that the expression of genes controlling the “atavistic” processes is usually kept under a transcriptional “brake” by genic or epigenetic modules typical of metazoan pluricellular organisms [45,46,47,48]. The idea is that those modules responsible for controlling processes typical of unicellular eukaryotes can de-emerge as a consequence of loss of control exerted by multicellular gene networks. Once these unicellular processes are unleashed, a differentiated eukaryotic cell, usually engaged in a structured tissue, starts to behave as a Proterozoic unicellular eukaryote, or as a yeast [48]. Hence, a set of “atavistic” markers are activated, the main one being lactic acid fermentation even in the presence of adequate O_2_ concentration: this is called aerobic glycolysis or the Warburg effect, after the German biochemist Otto Warburg discovered it in the 1920s [49,50]. The Warburg effect allows tumor cells to produce 2 ATP/1 Glucose but at least 10 times faster than OXPHOS [51], which instead provides 32 ATP/1 Glucose [52]. Additional hallmarks of cancer which can be explained with the atavistic model include: (a) dedifferentiation; (b) general reprogramming of energy metabolism; (c) limitless replication; (d) resilience to cell death; (e) insensitivity to growth suppression; (f) evading immune system; and (g) metastatic spread [53,54]. Indeed, the atavistic theory can provide convincing scientific coherence in supporting and explaining the hallmarks of cancer listed above. Regarding the possible triggers involved in the activation of the “atavistic” program, it has been suggested that normal eukaryotic cells under a challenging and “hostile” microenvironment can activate the “atavistic” program as a way to increase chances of survival. This means losing the “altruistic” and cooperative behavior which characterizes differentiated tissues and acquiring a “selfish”/“egoistic” behavior typical of unicellular organisms. Several stimuli have been proposed and shown to promote atavistic tumor transformation, such as chronic inflammation, oxidative stress, hypoxia and persistent exposure to radiation, chemical carcinogens, and infectious agents [55]. These stimuli can in turn strengthen the inflammatory milieu, which, in combination with local Fe^2+^ accumulation, would promote the cytosolic Fenton reaction, leading to OH- and •OH-production. It has been suggested that this alkalinization could drive a shift to lactic acid fermentation in order to buffer cytosolic alkalinization [55]. Indeed, it has been suggested that persistent disruption of the intracellular pH by Fenton reactions may be responsible for driving cancer development. Moreover, cancer cells can find a metabolic advantage from a sustained Fenton reaction at both the cytosolic and mitochondrial levels [56]. In particular, the Warburg effect could be explained by the fact that the Fenton reaction supports anaerobic respiration and glycolytic ATP synthesis. Therefore, ATP production can be triggered by Fenton reaction-sustained anaerobic respiration and consumption of mitochondrial protons, and thus the cells may have a very limited need for aerobic respiration [56]. This hypothesis has been recently confirmed and reviewed to underline its importance in tumor biology and the connection with the Warburg effect [57].

Therefore, we may extend the idea of a mechanism of “stress-induced mutagenesis” [58], according to which eukaryotic cells under a stressing/challenging microenvironment would undergo a “cellular maladaptation” process, which would in turn increase “waves” or “storms” of genomic instability [58].

In addition, as we will see in more detail in the following paragraphs, the metabolic rewiring of cancer cells can support an epigenetic rewiring, mainly through histone and non-histone lactylation, strengthening the tumor phenotype. For example, proliferating cancer cells need to shield themselves from apoptosis, the immune system, and other factors that may reduce their survival, such as contact-inhibition restraint or cell–cell competition in a growing tissue. Mutations in tumor suppressor genes might have been selected to help overcome these obstacles and improve the fitness and survival of cancer cells [56].

In conclusion of this paragraph, it is important to mention that metabolic rewiring can lead to a direct activation of mitogenic cell signaling pathways, such as the ERK pathway, promoting hepatocellular carcinoma (HCC) development. Moreover, using classical chemotherapeutics such as the multi-kinase inhibitor sorafenib in such dysmetabolic conditions promotes proliferation by further strengthening metabolic rewiring, especially glycolysis. Serine also accumulated, partially contributing to pERK activation [59].

Table 3 provide a synoptic overview of the main characteristics of the “atavistic” and metabolic theories of cancerogenesis.

### 4.2. Tumor as a Complex Metabolic Disease

Interesting questions now are: (i) How is the Warburg effect activated? (ii) What is the link between the onset of the Warburg effect and the genomic instability observed in most cancers? (iii) What is the link between the Warburg effect and the “atavistic” model for tumorigenesis described above? About point (i), several hypotheses have been made in the course of the time to explain the origin of the Warburg effect. According to Warburg himself, and several other biochemists later on, one main trigger should be recognized in mitochondrial damage [60,61]. Indeed, in several cancers, mitochondrial damage has been observed, and a link to the onset of the Warburg effect has been proposed [60,61]. In this respect, evidence has been brought that introducing healthy mitochondria from non-cancerous cells into highly malignant breast cancer cells was sufficient to dampen the malignant behavior and to decrease many of the cancer’s hallmarks, such as unregulated cell growth, survival under hypoxic conditions, escape from apoptosis, drug resistance, invasiveness and tumorigenicity in mice. Drug sensitivity was also improved [62]. Similarly, the tumorigenic potential of aggressive 143B osteosarcoma cells was much lower in cybrids containing normal mitochondria and tumoral nuclei, if compared with cybrids harboring normal nuclei and tumor cell cytoplasm containing mitochondria with structural abnormalities and mtDNA damage [63]. Also, it has been recently reported that restoring mitochondrial quality and quantity reverted the Warburg effect and promoted differentiation in neuroblastoma [64].

Having established some standpoints about topic (i), regarding topic (ii) several pieces of evidence can confirm that the stability of the nuclear genome, and hence the onset of somatic mutations, is linked to the proper functioning of the mitochondrial machinery. Indeed, it has been shown that the efficiency of mitochondria is essential for the maintenance of the integrity of the nuclear genome [61]. In addition, constant mitochondrial stress has been linked to promotion of lactic acid fermentation and error-prone/unfaithful DNA repair mechanisms [65,66,67,68,69,70]. In malignant melanoma cells, mitochondrial damage and the consequent impairment of oxidative phosphorylation (OXPHOS) lead to upregulation and addiction to aerobic glycolysis, together with activation of the BRAF oncogene [71]. Interestingly, aneuploidy has been shown to arise in cells where calcium (Ca^2+^) homeostasis was impaired due to loss of mitochondrial functionality [61]. Thus, we can say that there is convincing evidence that dysfunctional OXPHOS can be considered a very important driver of tumorigenesis. The consequent metabolic rewiring promotes a pro-mutagenic condition, which strengthens tumor phenotype. Indeed, Warburg himself stated: “Just as there are many remote causes of plague, heat, insects, rats, but only one common cause, the plague bacillus, there are a great many remote causes of cancer, tar, rays, arsenic, pressure, urethane, but there is only one common cause into which all other causes of cancer merge, the irreversible injuring of respiration.” In this context, mitochondria should take the stage as the main driver of tumorigenesis. For example, a defective or low-efficiency OXPHOS has been correlated with tumorigenesis in glioblastoma multiforme (GBM) [61,72]. Of note, it is important to point out here that there is a difference between OXPHOS and mitochondrial respiration. In many tumors, mitochondrial respiration is intact, but OXPHOS is impaired. This has led to some misunderstanding in the recent literature about tumor metabolism. Indeed, respiration is not the same as phosphorylation: most cancer cells use respiration for biosynthetic and redox homeostasis purposes, without functional OXPHOS [73]. This also explains the apparent dichotomy between Warburg’s theory and Weinhouse’s rebuttal [74]: they were just describing two sides of the same complex effect. In addition, we can say that the metabolic complexity and heterogeneity of cancers can further provide an explanation of the apparent divergence of Warburg’s and Weinhouse’s hypotheses. In fact, it is also true that high glucose levels can trigger the activation of fermentative glycolysis without preliminary mitochondrial damage, which is called the Crabtree effect. High glucose levels can also lead to functional mitochondrial damage and mitochondrial DNA damage, further strengthening the metabolic rewiring of cancer cells. We may perhaps introduce the idea of a “Warburg– Weinhouse effect”.

One important consideration is the following: all of the known pro-cancerogenic stimuli, such as chemical carcinogens, ionizing and ultraviolet radiation, viruses, intermittent or chronic hypoxia, inflammation and also some rare germline mutations, lead to an impairment of the cell’s respiratory efficiency through mitochondrial damage [61]. Another point of interest is that, so far, almost all tumor cells have been found to have defects in cardiolipin [61]. Cardiolipin is essential to maintain the efficiency of OXPHOS, because it is involved in the assembly and proper functioning of ETC supercomplexes [75]. Interestingly, we found, in one of our experimental works, that a common damage signature in cardiolipin characterized cancer cells upon acquisition of drug resistance [76].

Of note, it has been demonstrated that a hypoxic environment triggers the expression of genes controlling glycolysis, hence promoting tumor onset. This effect is regulated by an axis involving a specific hypoxia-sensitive miRNA (miRNA-548a-3p) and a specific transcription factor (SIX1). Thus, hypoxic conditions inhibit miRNA-548a-3p and the consequent activation of SIX-1 de-represses the biosynthesis of several key glycolysis markers such as LDHA, PKM2, ALDOA, PFKL, HK2, PGK1 and GLUT1 [77]. Interestingly, it has recently been shown that placing non-tumoral breast cells in a harsh microenvironment was sufficient to induce a transition to a Warburg phenotype, without involvement of somatic mutations [78].

**Figure 2:**
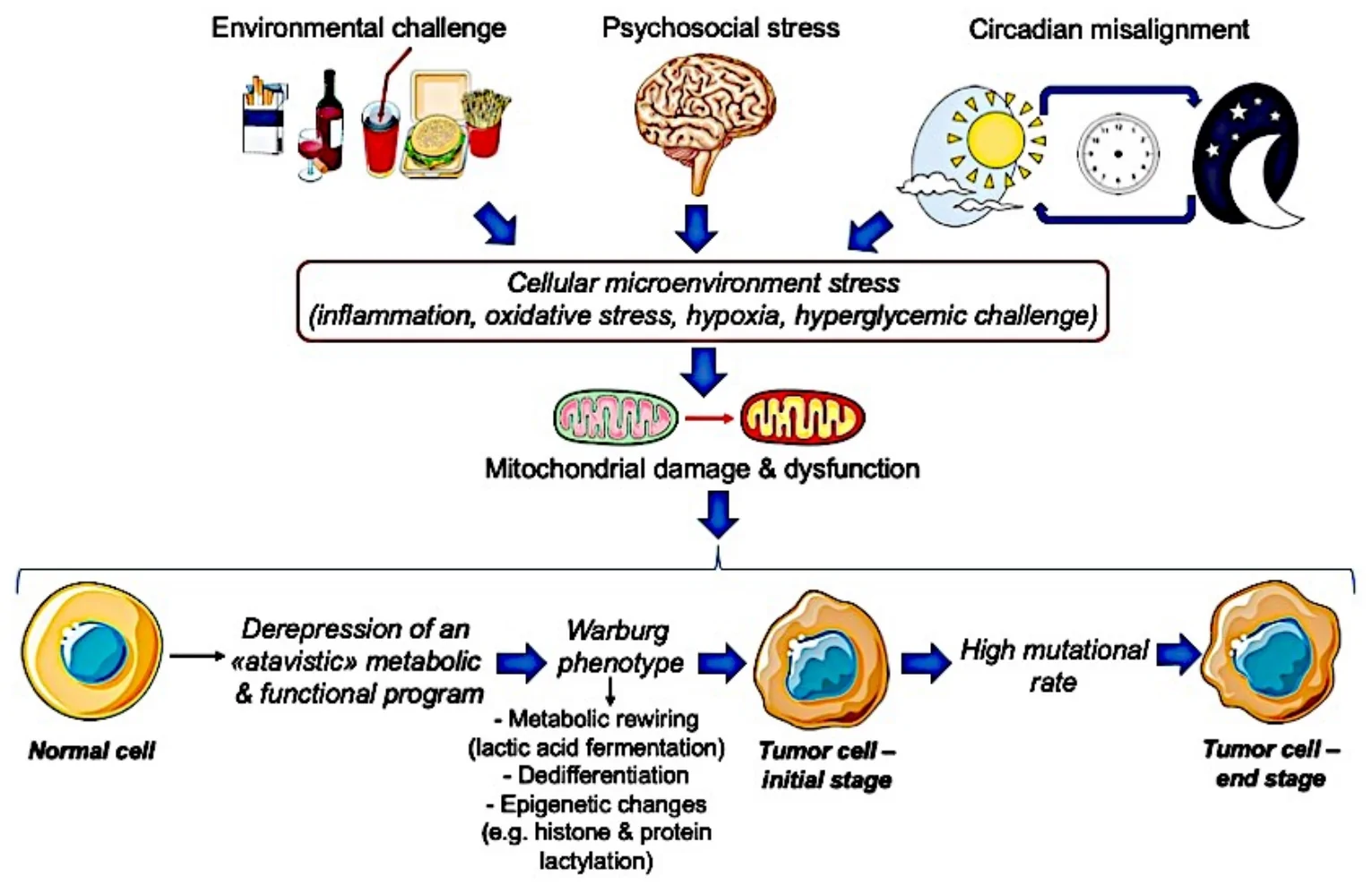
Graphical description of the biological complexity behind tumorigenesis.

Figure 2 summarizes ideas and scientific evidence about the complex/multifactorial/metabolic origin of cancer.

### 4.3. Metabolism, Epigenetics and Tumors

The central role of the metabolic–cellular axis in controlling cellular homeostasis, and, consequently, tumorigenesis, has a solid connection to epigenetics, which is the mirror of metabolic status on gene expression. In general, epigenetics has a very important role in the regulation of cellular function, probably more relevant than somatic mutations. A good example is provided by the onset of metaplasia, one of the mechanisms by which adult somatic differentiated cells adapt to mutated and more aggressive microenvironmental conditions. As an example, we can mention two types of metaplasia: (a) Barrett’s esophagus, in which squamous epithelial cells are replaced by intestinal-like columnar cells; and (b) squamous metaplasia, where columnar respiratory cells transdifferentiate into stratified squamous epithelium. It is worth underlining that both these types of metaplasia take place without any somatic mutation. Instead, a general reprogramming of gene expression is observed, driven by activation of specific transcription factors (TFs) and epigenetic changes. For (a), there is upregulation of the TF CDX2 [79], and for (b), of the TF p63. Regarding the role of epigenetics, this has been acknowledged in some other examples of metaplasia, such as the switching of B lymphocytes into macrophages or transformation of exocrine pancreas to hepatocytes [79]. Recently, the well-known predisposition to the onset of gastric cancer (GC) from incomplete-type gastric intestinal metaplasia (GIM) (Inc IM) has been shown to be correlated with the degree of methylation of specific genes. In particular, Inc IM organoids showed bipotent differentiation on bone morphogenetic protein (BMP) stimulation. Epigenetically, Inc IM exhibited diffuse intergenic hypermethylation with a methylation pattern more closely resembling native antral mucosa. In contrast, complete IM was characterized by promoter hypermethylation of tumor suppressor genes and a more fully “intestinal” epigenetic profile. Also, spatial mapping identified Inc IM-like cells in a subset of GCs, particularly the microsatellite stable subtype, indicating a phenotypic continuum from Inc IM to GC [80].

This last point gives us the right theoretical background to discuss how epigenetics, metabolism, the Warburg effect and tumorigenesis are linked. In fact, a connection has been described and plays a very important role. The term “epigenetics” refers to all those modifications of gene expression not directly due to gene mutations or gross chromosomal changes or rearrangements. Such gene expression changes are mediated by DNA methylation and chemical modifications of histones, namely methylation, acetylation, lactylation, crotonylation, ubiquitylation, phosphorylation, SUMOylation, and ADP-ribosylation [81]. In addition, less common forms of histone modifications have been reported, such as propionylation, butirylation, hydroxylation and formylation [81]. These chemical modifications take place at the level of five classes of histones: H1, H2A, H2B, H3, and H4. Each nucleosome includes a core octamer formed by two copies of each of the four core histones, H2A, H2B, H3 and H4, while H1 binds to the DNA between nucleosomes, in this way compacting the chromatin. Of note, histones H3 and H4 possess nucleosomal tails that can be covalently modified at various sites [82]. It is worth spending some words on lactylation, since it has recently been extensively investigated, with several very important outcomes. As we pointed out in the previous paragraphs, increased lactate production is a hallmark of all tumor cells, due to the Warburg effect. Indeed, after the first discovery in 2019 [83], several points have been clarified. For example, it has been proposed that increased levels of lactate observed during the process of tumorigenesis can contribute to the progression, establishment and strengthening of the tumor phenotype. In particular, in the early stages, lactylation of p53 by the enzyme alanyl-tRNA synthetase 1 (AARS1) supports tumorigenesis by impairing its function and translocation to the nucleus [84].

About the role of epigenetics in the onset/regulation/control of the Warburg effect several interesting pieces of evidence have been collected during the last decade which contributed to increasing our awareness on how the Warburg effect is triggered and regulated; however, many points still await clarification. Concerning the role of methylation, it is known that DNA methylation is involved in controlling the expression of several important glycolytic enzymes, such as LDH, PKM2, HK and GAPDH. In addition, DNA methylation has been correlated with mitochondrial dysfunction observed in tumor cells. Of note, activation stimuli for these pathways seem to lie in the need for rapid ATP biosynthesis, increased need of biosynthesis intermediates and the need to decrease ROS production. It has also been proposed that hypoxia and an acidic microenvironment can trigger this methylation step. Still, how microenvironmental stress can trigger DNA methylation is still to be defined. Interestingly, an epigenetic mechanism bridging the metabolic rewiring that characterizes the Warburg effect and the onset of dedifferentiation typical of tumors has been suggested. According to this view, mitochondrial impairment or damage, which is typically observed in tumor cells, triggers a metabolic adaptation, leading to ATP production mainly by aerobic glycolysis and production of lactate from pyruvate. This lactate synthesis has the purpose to regenerate NAD^+^, and is mainly driven by the excess of NADH that typically characterizes tumor cells. Still, tumor cells retain a low NAD^+^/NADH ratio if compared to healthy cells: this is called reductive stress. Reductive stress is, in turn, responsible for driving several metabolic changes: (a) α-ketoglutarate (α-KG) is converted to L-2-Hydroxyglutarate (L-2-HG); (b) reductive carboxylation; and (c) de novo proline biosynthesis. In particular, L-2-HG inhibits several α-KG-dependent dioxygenases, such as ten-eleven translocation (TET) DNA demethylases, Jumanji domain-containing histone demethylases (JMJDs), and prolyl hydroxylases (PHDs) [85]. These changes promote DNA and histone hypermethylation, as well as upregulation of HIF, resulting in cell dedifferentiation [86]. Moreover, metabolically rewired tumor cells produce less Ac-CoA, due to the impairment of mitochondrial function [87]. Two examples can be cited: (a) in neuroblastoma cells, hypoxia leads to a HIF-1α-mediated activation of pyruvate dehydrogenase kinases (PDK1/PDK3) that, in turn, inhibits pyruvate dehydrogenase (PDH), decreasing endogenous acetyl-CoA production, leading to histone hypoacetylation and inhibition of differentiation. Interestingly, acetate addition reverted the process, even under hypoxic conditions [88]; (b) in breast cancer cells, serine deprivation inactivates PKM2, thus decreasing intracellular pyruvate and Ac-CoA. The consequent histone hypoacetylation leads to the downregulation of estrogen receptor (ER) and progesterone receptor (PR), predisposing to the onset of the ER-/PR- breast cancer, which is highly resistant to the current therapeutic protocols [89]. It has also been recently underlined the importance of epigenetic dysregulation in the progression of pancreatic cancer and pancreatic ductal adenocarcinoma (PDAC), and also in the process of metastasis and immune evasion [90].

### 4.4. Metabolic Disorders, Stress, and Tumor Development

Growing evidence is being accumulated about the triggering effect on tumorigenesis exerted by metabolic disorders, and, in particular, by insulin resistance/T2DM and by metabolic syndrome sensu lato. Indeed, the correlation between dysmetabolism, T2DM and tumor development is being increasingly investigated. In addition, the importance of the association between psychosocial stress, onset of metabolic diseases and cancerogenesis is likewise getting more attention. Let us consider some examples. A study performed in mice showed that a diet rich in refined carbohydrates led to increased body weight, plasma insulin and IGF-1 levels, and increased xenograft tumor growth of LNCaP human prostate cancer cells. Moreover, these tumors also showed high levels of phosphorylated Akt (activated) and slightly increased expression of the insulin receptor. Interestingly, serum from mice on the high carbohydrate–high fat diet was more mitogenic than serum from mice fed the low carbohydrate–high fat diet [91]. In general, hyperinsulinemia and the overactivation of the insulin signaling pathway have been correlated with poor prognosis and reduced survival in breast cancer [92,93]. In breast cancer, T2DM and metabolic syndrome have also been associated with increased incidence and adverse prognosis [94]. In the extremely aggressive Triple-Negative Breast Cancer (TNBC), growing evidence supports the promoting role of metabolic syndrome [95]. Mechanistically, hyperinsulinemia has been found to promote TNBC by increasing histone acetylation on promoters of insulin-induced genes, thus triggering TNBC, increasing uncoupled mitochondrial activity and decreasing superoxide dismutase 2 (SOD2) expression, thereby predisposing to genomic instability [96].

In general, from an epidemiological point of view, hyperinsulinemia, obesity and T2DM have been associated with the onset of many tumors [97]. Interestingly, hyperinsulinemia has been found positively associated with tumor mortality independently of diabetes, obesity, and metabolic syndrome [98,99]. It is worth mentioning here the role of “exposome” or “envirome” as an important determinant/promoter of the so-called chronic non-communicable diseases (CNCDs), such as diabetes, obesity, and cancer. “Exposome”/“Envirome” refers to all of the stimuli with which people get in touch, stimuli whose effects they have to counteract [100]. The central issue when talking about the negative effects of stress is that, as human beings who belong to the animal kingdom, we are predisposed to deal with acute types of stressful events, typically defined as the “fight or flight” reaction [101]. However, when the stress gets chronic, it can become very dangerous and set the stage for the onset of metabolic diseases that, in turn, can favor tumor development [102]. Such unnatural resilience to which we expose ourselves in the very stressful “anthropocene” environments is not without consequences. Indeed, such psychological stress can activate neuroendocrine circuits, such as the hypothalamus–pituitary–adrenal (HPA) and sympatho–adrenomedullary (SAM) axes, and the autonomic nervous system. Chronic stress can also lead to insulin resistance by inhibiting the GH-IGF-1 axis, dampening thyroid function, and decreasing energy metabolism. In addition, increased hepatic glucose production, disrupted sleep patterns and circadian misalignment, as we will see in the next paragraph, hyperlipidemia, and a chronic/maladaptive pro-inflammatory environment can predispose to tumor onset. Mechanistically, the activation of the SAM, HPA and autonomic nervous system leads to overproduction of catecholamines and glucocorticoids [103]. Raised levels of glucocorticoids can impact tumor development in several ways: in breast cancer, glucocorticoids can promote tumorigenesis and metastasis [104,105], as well as dedifferentiation and drug resistance [106] by activating specific transcription factors. About catecholamines, there are now several pieces of evidence which support the involvement of adrenaline and noradrenaline in promoting and sustaining the process of carcinogenesis. For example, in breast cancer cells, a degradation of TP53 and an increased expression of *LDHA* have been reported, together with an anti-apoptotic action, which has been observed also in prostate cancer cells [107]. Also, stress hormones can trigger the upregulation of pro-inflammatory pathways, such as COX-2/PGE2, which strengthen the inflammation in the microenvironment, further worsening tumor progression and dampening immune response [108,109]. The metabolic phenotype characterizing obesity has been found to predispose to tumorigenesis, in particular, increased levels of (i) insulin, (ii) IGF-1, (iii) leptin [110]. Regarding diabetes, the hyperglycemia that characterizes T2DM can predispose to tumorigenesis and support cancer progression in several ways: (i) excess glucose can per se promote the shift between OXPHOS and fermentative glycolysis by triggering the Crabtree effect [111]; (ii) chronic hyperglycemia leads to the production of Advanced Glycation End Products (AGEs), which can damage mitochondrial membranes and dampen mitochondrial efficiency [112]; and (iii) inflammation-mediated ROS production can damage mitochondrial DNA, impairing function [112].

Of note, the consumption of ultra-processed food has been found to promote tumor development [113], and diabetes-induced hyperglycemia boosts primary tumor progression by promoting extracellular matrix stiffening due to glycation [114]. Importantly, the role of chronic stress in promoting tumor onset and development has been demonstrated in breast cancer.

Interestingly, the positive correlation between stress, depressive states, immune system function and tumor development was underlined more than 20 years ago in a *Lancet Oncology* paper [115]. Also, dopamine has been shown to block ovarian carcinoma growth triggered by high stress conditions [116]. In breast cancer patients after surgery, beta-adrenergic and COX2 inhibition improved post-operative outcome and recurrence [117,118]. Chronic psychological stress has been shown to boost lung metastasis of circulating breast cancer cells by promoting beta-adrenergic signaling [119]. Of relevance, chronic stress has been shown to accelerate and strengthen UV-triggered skin carcinogenesis [120].

Finally, it is worth highlighting here that, between 2015 and 2018, an important concept was formulated, namely that psychological stress and mitochondrial function are deeply interconnected. Indeed, chronic and intense psychological stress can translate into structural and functional mitochondrial impairment, defined as “Mitochondrial Allostatic Load (MAL)”. This, in turn, can be responsible for the onset of CNCDs, including cancer. So, mitochondrial damage induced by psychosocial stress is central in driving the onset of CNCDs, and, in addition, mitochondrial impairment can further worsen the levels of the biochemical mediators of stress [121,122,123,124,125]. Of note, a 2021 study performed in mice demonstrated that exposure to early-life stress (ES) increases the incidence of metabolic diseases and obesity, and consequently, other CNCDs and cancer. The authors suggest that ES negatively affects mitochondrial function throughout life, underlining the central role of mitochondrial integrity and proper functioning in the prevention of CNCDs [126].

### 4.5. Circadian Rhythms Misalignment, Metabolism and Tumors

**Figure 3:**
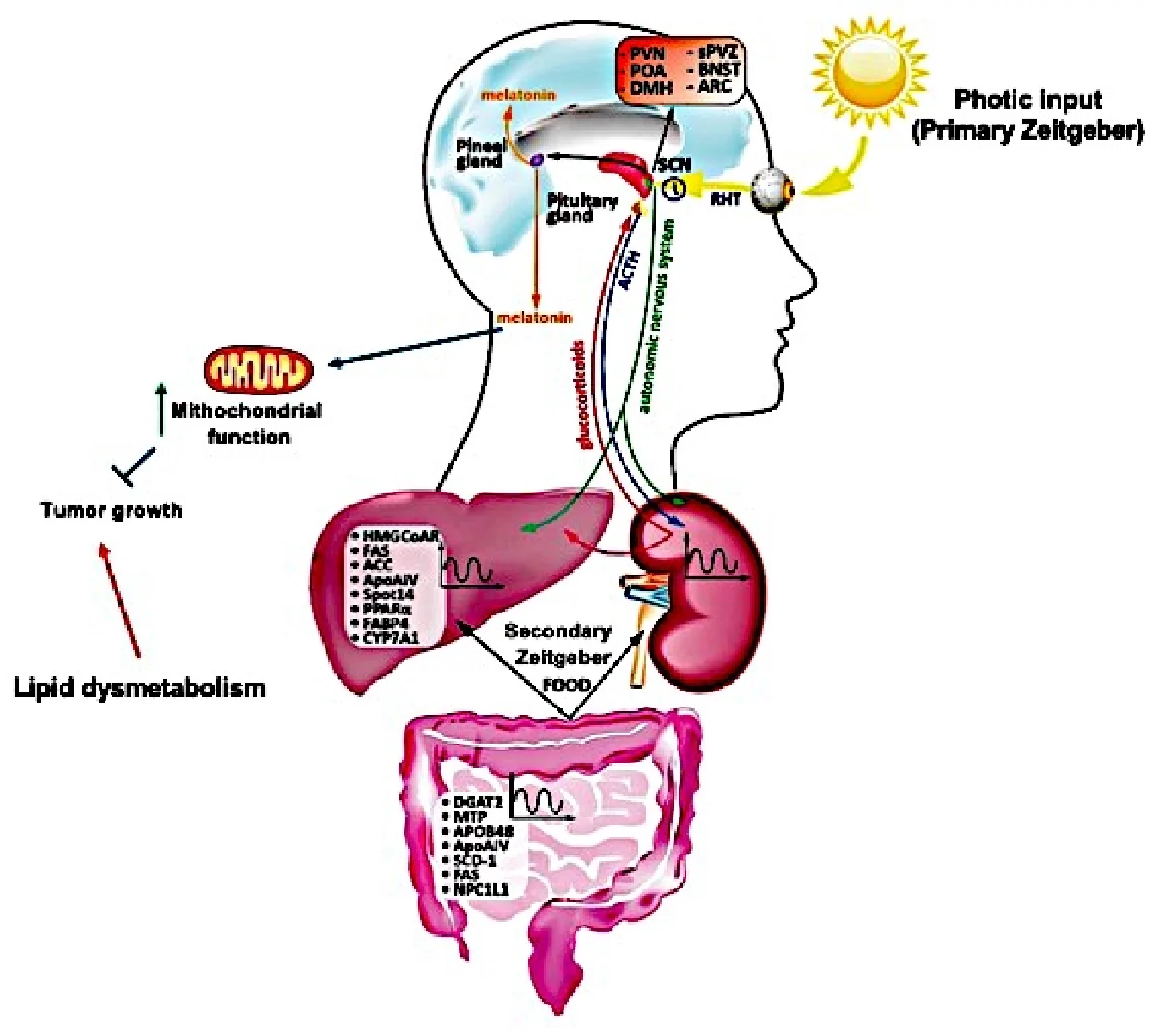
Graphical summary of the regulation of circadian rhythms and their role in lipid dysmetabolism and tumorigenesis. Modified from Gnocchi D. et al., *Biology*, 2015 [133].

Another important topic is the involvement of circadian rhythm misalignment in metabolic dysregulation and, consequently, how this affects tumor onset and development. An extensive revision on the mechanisms at the basis of the correct functioning of circadian clock machinery is out of the scope of this review. Still, some pillars need to be elucidated. Circadian clocks are centrally orchestrated in the brain by the Suprachiasmatic Nucleus (SCN), a small structure of about 10,000 neurons on each side of the third ventricle in the anterior part of the hypothalamus, above the optic chiasm. When the SCN is isolated in organotypic culture, its autonomous timing mechanism keeps working indefinitely, with precision and robustness [127]. The photic projections are the retinohypothalamic tract (RHT) from retinal ganglion cells and the geniculohypothalamic tract (GHT) from the intergeniculate leaflet (IGL). The RHT gets inputs from photosensitive ganglion cells in the retina. Then, RHT secretes glutamate into the core SNC VIP regions, in this way, mediating the photic regulation of circadian rhythmicity. The GHT provides an accessory photic input mediated by both photic and non-photic stimuli, such as motor activity [128]. In the SCN, the GHT and the RHT partially overlap in their innervation. Neurotransmitters involved in the GHT include neuropeptide Y (NPY), GABA, and enkephalin (ENK). NPY acts pre-synaptically to inhibit GABA-mediated transmission to the SCN [129]. The median raphe nuclei located in the midbrain produce serotonin and release it into retinorecipient regions of the SCN, specifically in VIP-containing regions. During the day, serotonin activates the circadian pacemaker by potentiating the response to glutamate; then, during the night, it inhibits this input. Serotonin regulates SCN neurons by both presynaptic and postsynaptic mechanisms [128]. In the hypothalamus, efferent SCN projections terminate at: (i) the medial preoptic nucleus; (ii) subparaventricular zone; (iii) ventromedial nucleus; (iv) dorsomedial nucleus; (v) ventral lateral geniculate nucleus; and (vi) lateral septal nucleus. In the thalamus, projections terminate in the anterior part of the paraventricular nucleus. Projections to the preoptic nucleus control sleep and wakefulness [130]. The major polysynaptic efferent of SCN neurons project to the pineal gland. During the night, these fibers release noradrenaline and stimulate postsynaptic β-1 and α-1 adrenergic receptors on pinealocytes, stimulating the production of melatonin [131,132]. The SCN uses humoral and neuronal inputs to coordinate circadian machinery in the peripheral organs such as: lung, liver, kidney, heart, and muscle [133]. These peripheral organs express circadian genes according to the combination of photic input to the SCN and non-photic stimuli such as the timing of food intake or motor activity [131,132]. Figure 3 summarizes some general concepts about circadian rhythms regulation and their role in mitochondrial function.

Three decades of research led to the identification of the molecular machine controlling the circadian clock. The transcription of the so-called “clock-controlled genes” is activated by the dimer formed by CLOCK⧫BMAL1, two bHLH proteins that bind to promoters containing E-boxes (CACGTG). In the forebrain, the role of CLOCK is performed by another transcription factor named NPAS2. The transcriptional machine is completed by the family of CRY transcriptional repressors that dimerize with PER (PER⧫CRY) to inhibit gene expression triggered by CLOCK⧫BMAL1 [134]. Interestingly, circadian rhythmicity can be kept even in the absence of transcription, as demonstrated in erythrocytes for the oxidation of peroxiredoxin proteins, which is entrained by environmental zeitgebers, such as temperature [135]. Thus, the full operativity of the circadian clock machinery seems to be the result of a close collaboration between nuclear and cytoplasmic components [136]. In general, there is a deep connection between the central clock, peripheral clocks, metabolism and tumorigenesis. Now it is known that circadian misalignment per se can trigger deregulation of the secretion of the peptides involved in the control of feeding, such as NPY, AgRP (Agouti-Related Peptide), in this way, increasing food intake. Moreover, stress stimuli, such as glucocorticoids, can signal to the SCN, changing its activity and, hence, shifting the expression of many metabolic enzymes in the peripheral organs [17,133]. Indeed, peripheral organs express the circadian machinery and metabolic enzymes are under circadian control [133].

The awareness of the pleiotropic importance of circadian rhythms led to increasing knowledge about the role of desynchronizing conditions, dysmetabolism and cancerogenesis. Although many things need to be pointed out and better investigated, some relevant conclusions can be drawn. First, a correct circadian alignment is involved in the control of many cellular processes. For example, sleep deprivation, or a condition of hypervigilance due to environmental stress, can lead to overproduction of corticotropin-releasing hormone (CRH) and, consequently, of ACTH, and increased production of cortisol. This leads to negative feedback, which reinforces stressful conditions, further aggravating the negative effects of cortisol, such as increased blood glucose and chronic inflammation. Moreover, stress-induced circadian misalignment induces an increase in ghrelin and a decrease in leptin, resulting in raised appetite, even without calorie deficit. In addition, the increased sympathetic activity together with hypercortisolism drives reduced glucose tolerance, hence predisposing to insulin resistance, obesity and metabolic syndrome, and, consequently, tumor onset [20]. Another important point is that circadian disruption leads to decreased production of melatonin, which has many important biological homeostatic roles, in particular those exerted at the level of mitochondria [137]. In fact, it has been shown that melatonin supports mitochondrial function at several levels: (i) increases the efficiency of OXPHOS; (ii) counteracts cardiolipin peroxidation, thus protecting ETC efficiency; (iii) scavenges ROS, protecting mitochondrial membrane integrity; and (iv) promotes disposal of damaged mitochondria by mitophagy [138,139].

In general, the picture that is emerging is that the circadian clock supervises and coordinates most of the processes involved in the maintenance of organismal homeostasis [140]. Therefore, a circadian “common denominator” groups metabolic disease, tumors and also aging. The molecular basis of this intricate web is being discovered and is under investigation. Still, several important points are now known: for example, the cellular homeostasis is strictly dependent upon the levels of NAD^+^, which activates Sirtuin1 that exerts pleiotropic beneficial effects, such as activation of mitochondrial biogenesis and activity, activation of autophagy and reduction in inflammation. NAD^+^ is produced by the enzyme nicotinamide phosphoribosyltransferase (NAMPT), whose biosynthesis is stimulated by CLOCK⧫BMAL1 [141,142]. In addition, it is worth mentioning that it has recently been reported that circadian misalignment can trigger tumorigenesis by directly enhancing glycolysis, probably through the loss of mitochondrial fitness and deregulation of genes involved in the control of glycolysis [143]. Likewise, HIF-1α expression was found to be under circadian control in hepatocellular carcinoma, and upon circadian disruption, an upregulation of GLUT1 and HK2 was observed, together with increased lactate production [144]. Of note, it has been suggested that melatonin could inhibit the Warburg effect by redirecting the oxidation of glucose to the mitochondria [145]. Moreover, it has been recently reported that in non-small-cell lung cancer (NSCLC) cells, melatonin effectively inhibits cell growth [146].

The role of circadian rhythms in the regulation of the immune system has recently been reported, showing that PER2 deficiency increased breast cancer progression by an immunosuppressive action [147].

### 4.6. Tumor’s Metabolism and Immunoescape

**Figure 4:**
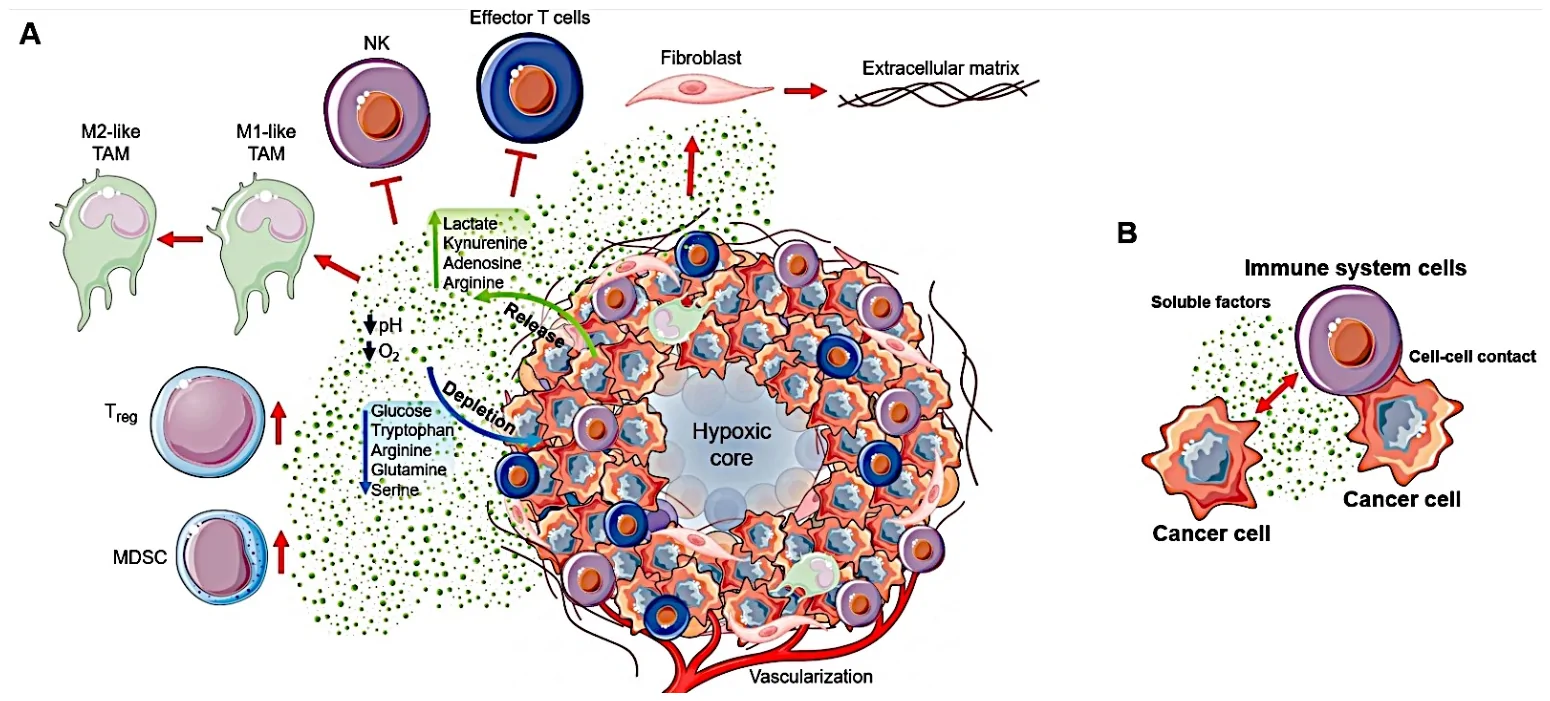
Schematic representation of the immunosuppressive TME and its major cellular interactions. (**A**) Hypoxia, nutrient depletion, acidic pH, and metabolic reprogramming contribute to immune dysfunction and tumor progression. Tumor cells consume essential nutrients, including glucose, tryptophan, arginine, glutamine, and serine, while releasing immunosuppressive metabolites, including lactate, kynurenine, adenosine and arginine, promoting MDSCs, Tregs, and M2 macrophages while impairing NK cell and M1 macrophage functions. M2 macrophages support angiogenesis, whereas cancer-associated fibroblasts remodel the extracellular matrix and restrict immune infiltration. Together, these processes establish an immunosuppressive niche that favors immune evasion and tumor growth. (**B**) Crosstalk between tumor cells and immune or stromal cells occurs through soluble mediators, extracellular vesicles carrying metabolites, microRNAs, proteins, and lipids, and direct ligand–receptor interactions. These interconnected mechanisms regulate immune activation, stromal remodeling, angiogenesis, and tumor progression.

Tumor cells adapt their metabolism to the surrounding environment, thus creating a TME characterized by critical nutritional deficiencies and the accumulation of immunoinhibitory metabolites. These conditions impair antigen presentation by dendritic cells (DCs), thereby limiting T-cell activation and suppressing the cytotoxic functions of cytotoxic T lymphocytes (CTLs) and natural killer (NK) cells. The abundance and functional state of tumor-infiltrating T cells may therefore have prognostic value. Moreover, immunosuppressive immune and stromal populations, including M2-like tumor-associated macrophages (TAMs), myeloid-derived suppressor cells (MDSCs), regulatory T cells (Tregs), and cancer-associated fibroblasts (CAFs), exploit their metabolic flexibility to adapt to the nutrient-deprived TME and cooperate in tumor immune evasion (Figure 4). Tumor metabolism shapes the TME by altering nutrient availability, metabolite composition, and physicochemical conditions such as hypoxia and acidification. These changes, in turn, reprogram the metabolism and function of immune cells, often promoting immunosuppressive phenotypes that compromise antitumor immunity. Thus, tumor-derived metabolites act as key mediators of local immune regulation and may also contribute to the establishment of systemic immune tolerance. Because tumor-infiltrating immune cells face an evolving TME, immunotherapy resistance cannot be attributed solely to tumor-intrinsic mechanisms. The TME includes malignant cells, immune populations, stromal cells, blood vessels, extracellular matrix components, and a wide range of soluble mediators that collectively influence therapeutic responses. On the one hand, tumor-derived metabolites, such as lactate, adenosine, and kynurenine, act as potent immunoregulatory signals that suppress antitumor immune responses. On the other hand, the depletion of key nutrients, including glucose, amino acids, and oxygen, limits the proliferation, survival, and effector functions of immune cells, thereby further impairing antitumor immunity.

#### 4.6.1. Tryptophan Depletion in the TME

One of the first mechanisms identified to explain tumor-induced immunosuppression was tryptophan catabolism mediated by IDO (indoleamine 2,3-dioxygenase), which degrades tryptophan into kynurenine. Earliest observations date back to the 1980s–1990s [148,149], while studies in the early 2000s demonstrated that IDO activity in tumors contributes to immune evasion, particularly from T lymphocytes [150,151]. IDO is expressed by many solid tumors, where its expression is frequently associated with poor prognosis and immune escape [152,153,154]. IDO-mediated tryptophan depletion in the TME inhibits DC maturation, thereby impairing their antigen-presenting capacity and their ability to activate T lymphocytes. This effect is mediated by activation of the amino acid stress sensor General Control Nonderepressible 2 (GCN2), activation of the Aryl hydrocarbon Receptor (AhR) by kynurenine metabolites, and indirect inhibition of mTOR signaling [154,155,156,157,158,159]. At the same time, mTOR inhibition reduces glycolysis in immune cells and can promote a tolerogenic phenotype characterized by reduced T-cell stimulatory capacity and enhanced Treg induction and expansion [160,161,162]. Activated DCs with high mTOR activity exhibit predominantly aerobic glycolysis, whereas mTOR inhibition promotes a shift toward oxidative phosphorylation (OXPHOS) [162,163,164]. Furthermore, since tryptophan is essential for the activation and proliferation of T lymphocytes and NK cells, its depletion via GCN2 leads to cell cycle arrest, impaired T lymphocyte proliferation and reduced pro-inflammatory cytokine production [154,155,165,166]. In addition, tryptophan deficiency, through GCN2 activation and mTOR inhibition, impairs macrophage activation and antigen presentation, while IDO-mediated tryptophan catabolism reduces CTL and NK-cell cytotoxicity, establishing an immunosuppressive TME [154,159,163,167]. In parallel, kynurenine and its downstream metabolites bind to and activate the AhR, altering gene transcription in immune cells. This signaling promotes the differentiation and expansion of Treg cells, suppresses CTL and NK cell function, and induces macrophage polarization toward the M2 phenotype, further strengthening an immunosuppressive microenvironment [158,168,169,170,171]. Moreover, IDO-derived kynurenine activates AhR signaling in MDSCs, enhancing their immunosuppressive activity and accumulation within the TME and reinforcing Treg-mediated suppression of antitumor T-cell responses [170,172,173,174,175,176,177].

Together, these mechanisms illustrate the close interconnection between tumor metabolism, the TME, and immune-cell metabolism and function.

#### 4.6.2. Arginine Depletion in the TME

This mechanism of metabolic immune suppression has been extensively characterized over the past two decades. Tumor-induced arginine depletion within the TME impairs the effector functions of CTLs and NK cells [178,179,180,181]. Activated T lymphocytes depend on extracellular arginine to sustain metabolic reprogramming, proliferation, and effector differentiation. Consequently, arginine deprivation impairs T-cell receptor (TCR) and mTOR signaling, cell-cycle progression, and cytokine production, eventually promoting dysfunctional or exhausted phenotypes [178,181,182]. The key enzyme responsible for this depletion is arginase-1 (ARG1), which hydrolyzes L-arginine into L-ornithine and urea, reducing the extracellular availability of this semi-essential amino acid. Accumulating evidence indicates that several tumor types, including hepatocellular carcinoma and colorectal cancer, can express ARG1 [183,184]. ARG1 expression by immunosuppressive myeloid cells, including MDSCs, TAMs, and tumor-associated neutrophils (TANs), is the best-characterized mechanism of arginine depletion in the TME and a major indirect driver of tumor-induced immune dysfunction [178,182,185,186].

#### 4.6.3. Glucose Deprivation in the TME

This represents one of the most extensively studied metabolic mechanisms underlying tumor immune escape. Due to their high glycolytic activity, tumor cells consume glucose at a significantly higher rate than surrounding cells, creating a glucose-depleted microenvironment that limits nutrient availability for infiltrating immune cells. Therefore, important antitumor immune populations become metabolically compromised. Glucose restriction impairs the effector functions of both CTLs and NK cells by reducing interferon-γ (IFN-γ) production, a cytokine essential for antitumor immunity [187,188]. Consistent with the dependence of NK-cell function on glucose metabolism [189], tumor-derived TGF-β induces FBP1 in NK cells in lung cancer, suppressing glycolysis and cytotoxicity [190]. On the other hand, glucose metabolism is fundamental for the activation of NK cells [189]; in fact, the inhibition of the transcription factor Sterol Regulatory Element-Binding Protein (SREBP) compromises the metabolic reprogramming induced by cytokines by reducing glycolysis and oxidative phosphorylation (OXPHOS), with a consequent decrease in IFN-γ production and NK cell cytotoxicity [191]. DCs require increased glycolysis to activate the stimulator of interferon genes (STING) pathway and sustain type I interferon (IFN-I) production; therefore, glucose scarcity attenuates DC-mediated antitumor immunity [192]. Glucose deprivation also promotes immune dysfunction through epigenetic reprogramming: in CD8^+^ T cells, it induces miR-26a and miR-101, which downregulate the enhancer of zeste homolog 2 (EZH2), reducing histone H3 lysine 27 trimethylation (H3K27me3) and IFN-γ and tumor necrosis factor (TNF) production [193].

These findings highlight the intricate interplay between cellular metabolism and epigenetic regulation in shaping antitumor immune responses and reveal how metabolic competition within the TME contributes to tumor immune evasion.

#### 4.6.4. Glutamine Deprivation in TME

This represents a common mechanism of metabolic immune evasion across diverse malignancies and has been documented in several solid tumors [194,195,196,197]. Tumor cells consume glutamine to sustain anabolism, bioenergetics, and redox homeostasis, thereby depleting it within the TME [194,195,196]. This metabolic competition restricts glutamine availability to infiltrating immune cells, particularly CTLs, impairing their proliferation, cytokine production, and cytotoxicity [194,195,197]. In parallel, glutamine deficiency promotes Treg function, supporting macrophage polarization into the M2 phenotype, and enhancing the suppressive activity of MDSCs [195,197,198].

#### 4.6.5. Serine Deprivation in the TME

Like glutamine, tumor cells exhibit a high demand for serine to sustain nucleotide, protein, and glutathione synthesis, redox homeostasis, and rapid proliferation, thereby depleting extracellular serine within the TME [199,200]. This metabolic competition restricts serine availability for infiltrating immune cells, particularly CTLs, which require serine to support proliferation, one-carbon metabolism, and effector functions [197,199,200,201]. Consequently, serine deprivation impairs T-cell expansion by limiting de novo nucleotide synthesis [202], IFN-γ production, and cytotoxic antitumor immunity [144,199,200]. Altered serine metabolism also contributes to an immunosuppressive TME by promoting Treg stability and M2-like macrophage polarization through metabolic and epigenetic mechanisms [197,200,203].

#### 4.6.6. Lactate Accumulation in TME

Results from enhanced glycolytic activity in tumor cells. Lactate-driven intracellular acidification and reduced ATP synthesis impair T- and NK-cell viability and inhibit the secretion of effector cytokines such as IFN-γ [204,205]. In murine melanoma and pancreatic cancer models, NK cells exposed to LDHA-deficient tumor cells, characterized by reduced lactate secretion, showed increased IFN-γ and granzyme B production and enhanced NK-cell-mediated tumor cytotoxicity [205,206]. In melanoma, tumor LDHA expression and lactic acid production inversely correlate with T-cell infiltration and function [205]. In colorectal cancer liver metastases, lactate-associated acidification lowers the intracellular pH of liver-resident NK cells, leading to mitochondrial ROS accumulation, mitochondrial dysfunction, and apoptosis [207].

Lactate can also suppress M1-like macrophage functions and promote M2-like polarization. Tumor-driven glucose depletion and lactate-associated acidification, coupled with the inability of the dysfunctional tumor vasculature to rapidly remove H^+^ ions, promote immune tolerance by causing tumor-infiltrating lymphocytes (TILs) dysfunction, suppressing cytokine production, and promoting the accumulation of tolerogenic cells [208,209]. These conditions are associated with the secretion of G-CSF and GM-CSF by local mesenchymal stem cells and the upregulation of tumor-derived colony-stimulating factors, which in turn promote MDSC infiltration. Lactate can also stabilize hypoxia-inducible factor HIF-1α-dependent signaling, promoting an M2-like macrophage phenotype [210]. In cell-line models, MDSC accumulation can be reduced by inhibiting glycolysis and consequently decreasing G-CSF and GM-CSF release [211]. Furthermore, increased glucose consumption can promote a Treg phenotype, whereas TLR8 signaling inhibits glucose metabolism, thereby reducing the suppressive function of Treg cells [212]. A lactate-driven epigenetic modification was first described by Zhang et al. [83]. In macrophages, lactate acts as an epigenetic regulator by inducing histone lysine lactylation (Kla), particularly histone H3 lysine 18 lactylation (H3K18la). Kla accumulates at the promoters of specific genes and promotes transcriptional activation, representing an epigenetic modification with regulatory functions. In activated M1 macrophages, progressive lactate accumulation induces H3K18la and promotes the expression of genes associated with tissue repair and an M2-like phenotype, such as *Arg1* and *Vegfa*, activating transcriptional programs that suppress the inflammatory response and favor an immunosuppressive phenotype in macrophages [83].

Following this pioneering discovery, mounting evidence has demonstrated that tumor-derived lactate promotes non-histone protein and histone lactylation, thereby reprogramming the tumor microenvironment through epigenetic and post-translational mechanisms. Specifically, non-histone lactylation of PD-L1 in colorectal cancer cells enhances its stability, suppresses CD8^+^ T-cell-mediated antitumor immunity, and promotes immune evasion [213], whereas lactylation of LRPPRC in tumor-associated macrophages (TAMs) of hepatocellular carcinoma drives glycolytic reprogramming and M2-like macrophage polarization, thereby fostering an immunosuppressive tumor microenvironment [214]. In addition to macrophages and tumor cells, recent studies have shown that lactate-mediated lactylation also impairs dendritic cell function by reducing antigen presentation, increases the suppressive activity of myeloid-derived suppressor cells (MDSCs), promotes the stability of regulatory T cells (Tregs) through the lactylation of non-histone proteins such as MOESIN, and suppresses the cytotoxicity of natural killer (NK) cells by disrupting their metabolic capacity and effector functions [215,216,217,218]. Regarding histone lactylation, in glioblastoma, glucose-derived tumor lactate induces H3K18 lactylation in monocyte-derived macrophages, stimulating the expression of immunosuppressive genes and impairing T-cell activation [219]. Similarly, in pancreatic ductal adenocarcinoma, lactate-dependent histone lactylation establishes a positive feedback loop that promotes M2 macrophage polarization and tumor progression [220]. Collectively, lactate-induced lactylation is a central epigenetic mechanism by which tumors suppress innate and adaptive immune responses to create an immunosuppressive microenvironment.

#### 4.6.7. Lipid and FFA Accumulation in TME

Tumor and stromal cells enrich the TME with free fatty acids (FFAs), oxidized lipids, and cholesterol metabolites, which are taken up by infiltrating immune cells through lipid transport and binding proteins, including CD36, FATP2, and FABPs, causing profound metabolic reprogramming. Excessive lipid uptake and fatty acid oxidation (FAO) promote M2-like TAM polarization and sustain MDSC suppressive activity, whereas lipid accumulation impairs DC antigen presentation and induces metabolic dysfunction and exhaustion in CD8^+^ T cells and NK cells [221,222,223,224,225,226,227]. Conversely, Tregs are adapted to use FAO and OXPHOS, allowing them to persist and maintain suppressive activity in the lipid-rich TME [223]. Furthermore, in pancreatic ductal adenocarcinoma, tumor-derived lactate-induced histone H3K18la upregulates ACAT2 transcription, reprogramming cholesterol metabolism and establishing a positive feedback loop that promotes macrophage-mediated immunosuppression and tumor progression [220]. This mechanism links glucose metabolism, epigenetic regulation, and lipid metabolism, illustrating the interdependence of metabolic pathways within the TME.

#### 4.6.8. Adenosine Accumulation in the TME

In tumors, chronic hypoxia, inflammation, and high rates of cell death promote extracellular ATP release, which CD39 hydrolyzes to AMP and CD73 converts to adenosine [228,229,230,231]. Tumor hypoxia further amplifies this pathway through the stabilization of hypoxia-inducible factor-1α (HIF-1α), which positively regulates the transcription of CD39, CD73, and adenosine receptors, sustaining adenosine accumulation in the TME [229,230,231,232,233]. Extracellular adenosine acts as a potent immunosuppressant by activating the high-affinity A2A receptor (A2AR) and low-affinity A2B receptor (A2BR). Signaling through these Gs-coupled receptors increases intracellular cAMP and activates protein kinase A (PKA), thereby suppressing multiple antitumor immune functions. A2AR and A2BR signaling in innate and adaptive immune-cell precursors also promotes differentiation toward immunosuppressive phenotypes [234,235,236,237]. Accordingly, the CD39/CD73-adenosine-A2AR axis is widely recognized as a metabolic immune checkpoint [229,230,231,233,237]. In the TME, adenosine impairs DC maturation and antigen presentation, limiting T-cell activation. It also suppresses CTL and NK-cell proliferation, IL-2, IFN-γ, and TNF-α production, cytotoxicity, and degranulation while promoting T-cell exhaustion. In macrophages, it promotes M2-like polarization and IL-10, TGF-β, and VEGF production. Adenosine also promotes Treg expansion, stability, and suppressive activity; CD39/CD73-expressing Tregs further increase adenosine production, establishing a positive feedback loop that reinforces immunosuppression [228,230,231,233,235,236,237].

For the correct understanding of the role of metabolism in the TME in the regulation of the cellular function of the immune system, it is important to consider that the metabolic state of immune cells determines their functional state, and therefore their effector or suppressive phenotype [238]. For example: (i) in effector T lymphocytes, glycolysis is essential for cytotoxic function [239]; (ii) in Tregs, OXPHOS and FAO support suppressive function [223]; and (iii) the activation of dendritic cells requires a switch to glycolysis, whereas predominantly oxidative metabolism is associated with a tolerogenic phenotype [240]. Indeed, the TME does not simply “inhibit” immune cells, but actively reprograms their metabolism through nutrient competition, hypoxia, and the accumulation of tumor-derived metabolites, thereby altering their function [238,239,240,241]. These observations identify cellular metabolism as a key regulator of antitumor immunity and support targeting metabolic pathways to inhibit tumor growth, restore immune function, and improve the efficacy of cancer immunotherapy.

## 5. Possible Translational Applications of the “Selfish Cell” Model

As we said above, the “selfish gene” model, with its theoretical background and practical application, did not lead to significant translational progress and concrete outcomes in clinics. Indeed, the many hopes and expectations in the possible translational outbreaks of the “Human Genome Project” have gone unfulfilled. No field of medicine has found a concrete and effective benefit from the knowledge provided by this genetic data. The only interesting information is related to the drawing of a more accurate evolutionary tree of the human species [5,6]. Hence, we can say that the “selfish gene” model is not functional for what concerns the interpretation of most diseases and possible translational applications. The problem is that complex diseases cannot be explained just by a gene-centric view, which is oversimplifying and reductionist. In general, it is not correct to think that there is a gene for this or a gene for that. Genes cannot control and determine complex phenotypes by themselves. So, there is not a gene for schizophrenia, or a gene/genes for tumorigenesis, or a gene/genes for type 2 diabetes mellitus or obesity. Genes are only “responders” to the inputs that the cell collects from the environment and translates into biochemical information, which, in turn, can modify, or not modify, gene expression. This change in paradigm may offer new effective perspectives in the management of CNCDs.

In this context, some examples can be mentioned: (i) for what concerns the therapy of cystic fibrosis, a combination of drugs targeting the cystic fibrosis transmembrane conductance regulator (CFTR), elexacaftor–tezacaftor–ivacaftor provided moderate benefits to patients (13.8% improvement of the expiratory volume) with mild side effects [242]. Still, despite the limited benefits, the costs of such therapies are very high [243]; (ii) in familial hypercholesterolemia, PCSK9 inhibitors, such as evolocumab and alirocumab has been found to be effective [244]. However, these drugs are expensive and several side effects have been documented, mostly infection-related and neurocognitive [245,246]. For what concerns tumor therapeutic applications, the polyclonal and adaptive nature of most cancers makes showing increased overall survival for many cancers very challenging. Moreover, the fraction of cancer patients whose tumors demonstrate clear evidence of “targetable” signatures is very small [11,12]. Likewise, for diagnostic purposes, the relevance of genetic variants in the diagnosis of cardiovascular diseases has been questioned [247]. The only few exceptions may be found in prenatal testing of rare diseases with a prevalent monogenic etiology [248,249]. Still, it is very important to underline that the pathological process should be considered as a complex and multilevel process, involving all of the many components of the organism. Indeed, a complex network of interactions between stroma, extracellular matrix (ECM) and epithelium is the basis of the building of healthy tissues. Hence, it is extremely difficult to deterministically predict the pathological phenotype of even a monogenic disease [250].

These examples underline the idea that complex diseases need complex, systemic, or “holistic” theoretical frameworks to be more completely and properly understood.

So, how could the “selfish cell” concept provide useful hints in the clinical management of tumors? One striking example is that concerning the so-called “tumor reversion” or phenotypic reversion of cancer. Indeed, after the first case described in 1907 by Askanazy about a spontaneous regression of a teratocarcinoma, a significant number of cases have been documented, either in vivo or in experimental models [251]. Notably, several in vitro studies support an epigenetic mechanism at the basis of tumor reversion [13,251]. Another example is the rising evidence that ketogenic diets are proving useful and effective in the management of cancer patients [252]. Indeed, many clinical cases have been reported about the efficacy of ketogenic diets in cancer patients [61,72]. In addition, approaches based on buffering the acidic microenvironment generated by cancer cells have been proposed and have been effective in reducing tumor cell growth and drug resistance [253,254,255]. For example, a combined protocol with proton pump inhibitors, ketogenic diet and anti-diabetic drugs has been proposed for multiple myeloma [256]. Also, recent approaches in patients treated with sodium bicarbonate or sodium citrate (alkalization therapy) proved effective in patients with non-small-cell lung cancer [257] and colorectal carcinoma [258]. Interestingly, the effectiveness of the alkalization therapy was independent of the mutation status of *EGFR*/*ALK* and *RAS*, respectively [257,258]. Likewise, recently, a sodium bicarbonate transdermal formulation (DYV800) buffered intratumoral pH, boosting anti-tumor immune responses in a murine model of bladder cancer [259].

Thus, a metabolic approach can prove useful and have fewer side effects compared to targeted or precision medicine pharmacogenomic approaches [7]. This evidence should be considered for making clinical protocols more effective and safer.

### Immune Response Recovery by Metabolism-Based Approaches

Growing recognition of the role of metabolic alterations in immune evasion is prompting increasing efforts to develop strategies that metabolically reprogram the TME. These approaches seek to restore nutrient availability, limit immunosuppressive metabolites, and improve immune-cell metabolic fitness, either alongside standard-of-care treatments or in combination with approved immunotherapies [194,260,261,262]. Among the metabolic targets currently under investigation, inhibition of IDO aims to prevent tryptophan catabolism, whereas ARG1 inhibitors restore arginine availability within the TME, thereby enhancing T-cell and NK-cell effector functions and improving the efficacy of PD-1-based immunotherapy. Similarly, blockade of the CD39/CD73–adenosine axis, through CD73 inhibitors or A2A receptor antagonists, reduces adenosine-mediated immunosuppression, while inhibition of monocarboxylate transporters (particularly MCT1) limits lactate accumulation and tumor acidosis, favoring immune cell activity. Targeting glutamine metabolism through glutaminase inhibitors further restricts tumor metabolic fitness while preserving nutrient availability for immune cells [194,229,230,263,264,265,266].

One representative example of the translational potential of TME modulation is provided by the recent pilot trial published in *Oncogene* [267], which investigated whether remodeling the TME could enhance the efficacy of immunotherapy. Following extensive in vitro and in vivo investigations, the authors translated their findings into a prospective pilot clinical study involving 30 patients with hepatocellular carcinoma (HCC) treated with anti-PD-1 therapy in combination with intra-arterial hepatic infusion of 5% sodium bicarbonate. This combinatorial approach showed preliminary and encouraging clinical activity. Although this 2026 study is distinct from the earlier TILA-TACE investigations, it should be considered within the broader clinical context of bicarbonate-based locoregional approaches in HCC. Previous TILA-TACE studies were limited by small patient cohorts, treatment crossover, and the absence of a demonstrated overall survival benefit [268]. Therefore, these preliminary results should be regarded as hypothesis-generating and suggest that local tumor alkalinization may modulate the immunosuppressive TME and potentially enhance the efficacy of PD-1 blockade in HCC, although confirmation in larger controlled studies is required [267].

This strategy may therefore represent an innovative therapeutic approach worthy of further investigation in malignancies sharing biochemical and immunological TME features with hepatocellular carcinoma, including cholangiocarcinoma, renal cell carcinoma, and pancreatic ductal adenocarcinoma. Among metabolic interventions currently under clinical investigation, targeting the IDO-mediated tryptophan catabolic pathway represents one of the most widely explored strategies. Several first- and next-generation IDO1 inhibitors, including indoximod, a tryptophan mimetic that counteracts the immunosuppressive effects of tryptophan depletion, linrodostat (BMS-986205/ONO-7701) and navoximod, both selective IDO1 inhibitors, as well as HTI-1090 (SHR9146), a dual inhibitor of IDO1 and tryptophan-2,3-dioxygenase (TDO), have demonstrated acceptable safety profiles and encouraging preliminary antitumor activity in early-phase clinical trials, particularly in combination with immune checkpoint inhibitors for the treatment of advanced solid tumors [269,270,271,272,273]. However, enthusiasm for this therapeutic approach has been dampened by negative results from the Phase III ECHO-301/KEYNOTE-252 study, in which the selective IDO1 inhibitor epacadostat, administered in combination with the anti-PD-1 immune checkpoint inhibitor pembrolizumab, failed to improve progression-free survival or overall survival compared to pembrolizumab alone in patients with advanced melanoma, highlighting the biological complexity of the kynurenine pathway and the limitations of targeting IDO1 as a single therapeutic strategy [269,270,274]. Nevertheless, clinical development of IDO-targeted therapies has continued, focusing on next-generation inhibitors, dual IDO1/TDO blockade to overcome compensatory kynurenine production, biomarker-guided patient selection, and rational combinations with immune checkpoint inhibitors. To date, more than eleven IDO/TDO-targeting agents have entered clinical evaluation, supporting the targeting of tryptophan metabolism as a strategy to enhance immunotherapy responses [269,270].

Also, arginase inhibition has emerged as one of the most promising immunometabolic strategies for restoring antitumor immunity by increasing the availability of L-arginine in the tumor microenvironment [275]. The first arginase inhibitor in this class, CB-1158 (INCB001158), demonstrated a favorable safety profile, effective target inhibition, and increased systemic arginine levels (proof-of-mechanism) [276] in Phase I/II clinical trials NCT02903914, although its antitumor activity as monotherapy was modest, supporting its use in combination with pembrolizumab [277]. More recently, second-generation dual ARG1/ARG2 inhibitors, such as OATD-02, have entered early-stage clinical evaluation (Clinical Trial NCT05759923) and may further enhance immune cell function by overcoming compensatory arginine catabolism [278,279].

Of note, in the first-in-human Phase I study NCT01791595, the selective MCT1 inhibitor AZD3965 was evaluated as monotherapy in patients with advanced solid tumors and diffuse large B-cell lymphoma or Burkitt’s lymphoma [280]. The study demonstrated an acceptable safety profile, despite dose-dependent retinal toxicity and the need for careful cardiac safety monitoring, together with clear target engagement and metabolic activity consistent with MCT1 inhibition, although antitumor activity as monotherapy was limited. These results suggested the clinical feasibility of targeting lactate metabolism and provided the rationale for combination strategies with immune checkpoint inhibitors to enhance antitumor immune responses [280]. Also, MCT1 inhibition emerged as a promising immunometabolic strategy, although further toxicity studies are necessary.

Table 4 summarizes several pharmacological agents discussed in the text and their mechanism of action.

Clinical translation of adenosine-targeted therapies has primarily focused on A2A receptor antagonists, which suppress T and NK cell activity. In the Phase I/Ib study NCT02655822, the selective A2A receptor antagonist ciforadenant (CPI-444) was evaluated as monotherapy or in combination with the anti-PD-L1 antibody atezolizumab in patients with metastatic renal cell carcinoma. The treatment was well tolerated and increased intratumoral CD8^+^ T-cell infiltration and broadened the circulating T-cell repertoire, supporting restoration of antitumor T-cell immunity, with the greatest benefit observed in patients with high adenosine gene expression, supporting biomarker-guided patient selection [281]. More recently, the dual A2A/A2B receptor antagonist etrumadenant (AB928) has been investigated in combination with immune checkpoint inhibitors and chemotherapy. In the Phase II ARC-9 (NCT04660812) trial, etrumadenant combined with zimberelimab (anti-PD-1) and mFOLFOX6 and bevacizumab improved overall survival compared with regorafenib in patients with metastatic colorectal cancer previously treated with oxaliplatin- and irinotecan-containing regimens. Although this finding should be interpreted in the context of regorafenib as the comparator, it further supports adenosine receptor blockade as a promising strategy to overcome metabolically mediated immune suppression [282].

Some immunometabolic approaches change immune cell metabolism to help immune cells resist the TME, instead of removing immunosuppressive metabolites. Pharmacological activation of AMP-activated protein kinase (AMPK) by biguanides, such as metformin and phenformin, and modulation of the mechanistic target of rapamycin (mTOR) pathway have been shown to improve CD8^+^ T cell fitness, promote memory differentiation, and enhance responses to immune checkpoint blockade in preclinical models. However, whether these specific immunometabolic effects translate into improved antitumor immunity and therapeutic efficacy in patients remains to be established. Because both AMPK and mTOR regulate multiple immune cell subtypes, including effector T cells and regulatory T cells, their therapeutic modulation requires careful optimization to maximize antitumor immunity while minimizing unwanted immunosuppressive effects [266,283,284,285,286,287,288,289,290].

Although most metabolic immunotherapies remain investigational, with only a limited number having reached early-phase clinical evaluation, accumulating preclinical and early clinical evidence supports the concept that metabolic reprogramming can convert an immunosuppressive TME into an immune-permissive environment, thereby potentiating existing immunotherapies rather than replacing them.

## 6. Discussion

At the present time, we are facing an alarming trend for the so-called chronic non-communicable diseases (CNCDs), which include, in addition to tumors, metabolic, cardiovascular, inflammatory and autoimmune diseases. Recent epidemiological data describe a picture whereby about 75% of deaths worldwide can be ascribed to CNCDs [source: www.ourworldindata.org]. Notably, the epidemiological trend of CNCDs is rising also in non-Western countries [16]. Interestingly, data reveal that behavioral risk factors, such as smoking, high alcohol consumption, unhealthy food, and insufficient physical activity, are primary drivers in the onset of CNCDs [291]. Also, it has been reported that more than 50% of deaths due to CNCDs can be prevented by lifestyle changes [291]. In fact, in the context of CNCDs, it has been evaluated that the increased prevalence of several cancers, including liver and pancreas, can be associated with high fasting plasma glucose [292]. For this reason, a deeper understanding of the basis of complex diseases is needed. In order to obtain such improved knowledge about CNCDs, we ought to change the perspective by which we approach the study of cell biology and its physiopathological translations.

Here, we reviewed the importance and the theoretical and translational implications of using the proper scientific paradigm for the understanding of chronic non-communicable diseases (CNCDs). In the last 6 decades, the scientific scenario has been dominated by the idea that a deep knowledge of genetics and molecular biology could provide most of the answers required for making steps ahead into translational approaches for the cure of CNCDs. Still, very ambitious projects, such as the “Human Genome Project,” did not lead to the hoped-for and expected benefits from the therapeutic point of view, and, overall, did not hold the many promises with which they began. Hence, a new and broader level of understanding is needed and, fortunately, a novel consciousness, even if slowly, is arising. Changing the paradigm will also help to change the perception of CNCDs in the general population, introducing the idea that more is in our hands, as individuals, in order to prevent and fight many diseases, including tumors. The lifestyle choices that are observed in Western countries today predispose people to the onset of CNCDs, with a minor role for genes as main drivers, but a more evident role of epigenetics [293]. Studies dating back almost 100 years already showed that wherever people do not follow a Western countries-like lifestyle, the incidence of CNCDs is very low, cancer included, even in people more than 80 years old [294]. Of course, these data must be considered with a prudential attitude; still, they can suggest some considerations. For example, the idea that cancer is increasing in Western countries due just to the increased lifespan should be reconsidered. The message is that reducing risk factors with a virtuous and healthy lifestyle can significantly improve the picture. Indeed, individual lifestyle and nutritional choices can make the difference for the prevention and cure of CNCDs, as reported and recommended by the WHO [https://www.who.int/activities/preventing-noncommunicable-diseases, accessed on 5 October 2026] as well. A confirmation of the importance of lifestyle, environmental and psychosocial factors can be found in the so-called “blue zones”, places in the world where there is a very high percentage of healthy ultra-centenarian people [www.bluezones.com]. So far, five places have been identified and demographically validated: Ogliastra (Sardinia—Italy), Okinawa (Japan), Nicoya (Costa Rica), Ikaria (Greece), and Martinique [295,296]. Studying the “exposome” and lifestyle choices in these zones can help reduce the burden of CNCDs we are currently experiencing [296]. What was observed in the “blue zones” is also in line with what was published 30 years ago in a paper which reported the results of a study performed in Denmark at the end of the 19^th^ century in twins, where the longevity was ascribed to the genetic background for only 20%, with the remaining 80% being ascribable to environmental factors [297].

In addition, a recent paper from *The Lancet* underlined the increased incidence in young people of several types of cancer in the last century, strengthening the importance of environmental risk factors [298]. The “Anthropocene era” in which we are currently living in Western and high-income countries, but increasingly also in developing countries, has created an unhealthy environment from many points of view, which predisposes to CNCDs [112]. Important roles have been attributed, for example, to ultra-processed foods [113], or to lack of vitamin D and physical activity [299].

In this context, cells become central in receiving, processing and conveying the inputs coming from the environment in a complex interplay, which still needs to be fully understood and disentangled. In this context, epigenetics can be considered a valuable candidate as a “translator” of microenvironmental stimuli to changes in cell function. In particular, it can act as a translator of metabolic rearrangements, a bridge between metabolism and gene expression. Thus, a hypothetical pathogenic axis can be suggested, in which environmental challenges change the inflammatory/redox homeostasis, which can in turn lead to a metabolic rewiring promoting cellular maladaptation at several levels, from mitochondrial damage to epigenetic changes. This, in the end, will lead to the various landscapes of CNCDs, still with a common environmental/dysmetabolic root.

Overall, the social environment we live in can change our internal milieu, which in turn affects the cellular microenvironment and consequently cell function. Understanding the way by which cell function is influenced and modified by stressors is challenging, but undoubtedly doable. Therefore, we believe that the time is ready to rediscover an important message already proposed in 1977 in a *Science* paper entitled “The Need for A New Medical Model: A Challenge for Biomedicine” [300], where a broader perspective in analyzing medical conditions is suggested.

## 7. Conclusions

After the discovery of the double helix structure of DNA, genetics and molecular biology dominated the scenes of biomedical research, leading to a wave of “gene-centrism”, which is still solidly ingrained in the way many scientists approach biological questions. Indeed, the “selfish gene” model is still widely considered a good paradigm to understand biological systems. However, several flaws are emerging in the actual efficacy of the gene-centric model in providing an effective theoretical and operational tool for the understanding and curing of CNCDs. For this reason, a change in paradigm is necessary in order to see biological systems in their complexity, and not just from a reductionist perspective. This change in paradigm is also necessary to put cells, and by extension tissue and organs, at the center of the analysis, considering genes just as one of the effectors of cell function. The cell says what to do to the genes, and not vice versa. Such a Kuhnian “revolution” is needed to better consider the complexity that characterizes living systems. This, in turn, will help in preventing and better managing physiopathological conditions, and in the improvement of the quality of life.

## Figures and Tables

**Table 1:** Examples of CNCDs and relative percentage of associated deaths according to the data obtained from www.ourworldindata.org.

CNCDs		
Disease	% of Deaths	Metabolic Root
Heart Diseases	33%	Lipid dysmetabolism
Tumors	18%	Damaged mitochondrial function
Chronic Respiratory Diseases	7%	Inflammation
Digestive Diseases	4.5%	Lipid dysmetabolism & inflammation
Neurological Diseases	3.9%	Brain insulin resistance
T2DM & “T3DM”	2.7%	Insulin resistance
Musculoskeletal Diseases & Chronic Kidney Disease	5.7%	Inflammation

**Table 2:** Comparison between somatic mutations theory (SMT) and Tissue Organization Field Theory (TOFT).

Theories		
Characteristics	SMT	TOFT
Core of disease	Cell	Tissue/organ
Mechanism	DNA mutations in single cell	Breakdown/impairment of cell–cell interaction
Default state of cells	Quiescence	Proliferation
Function of mutations	Main drivers of tumorigenesis	Secondary consequences of tumorigenesis
Sum up	Cancer as a genetic disease	Cancer as a tissue disorganization disease

**Table 3:** Comparison between the “atavistic” theory of cancer and the metabolic theory of cancer.

Theories		
Characteristics	Atavistic	Metabolic
Core of disease	Cell	Cell
Mechanism	Reactivation of a unicellular metabolic/proliferation program	Damaged mitochondrial function
Default state of cells	Controlled proliferation	Controlled proliferation
Function of mutations	Secondary consequences of tumorigenesis	Secondary consequences of tumorigenesis
Sum up	Cancer as a maladaptive cellular disease	Cancer as a mitochondrial disease

**Table 4:** Pharmacological agents discussed in the text and their principal mechanisms of action.

Pharmacological Agent or Regimen	Main Mechanism of Action
Trastuzumab	Humanized anti-HER2 monoclonal antibody
Bevacizumab	Humanized anti-VEGF monoclonal antibody
Atezolizumab	Humanized anti-PD-L1 monoclonal antibody
Ciforadenant (CPI444)	Selective A2A receptor antagonist
Etrumadenant (AB928)	Dual A2A/A2B receptor antagonist
Pembrolizumab	Humanized anti-PD-1 monoclonal antibody
Zimberelimab	Humanized anti-PD-1 monoclonal antibody
Metformin	Biguanide inhibitor of NADH: ubiquinone oxidoreductase, also known as complex I of the mitochondrial respiratory chain and indirectly activates AMPK
mFOLFOX6	Chemotherapy protocol consisting of oxaliplatin, folinic acid (leucovorin), and 5-fluorouracil (5-FU)
Regorafenib	Multikinase inhibitor that targets multiple kinases involved in tumor angiogenesis (including VEGFR1–3 and TIE2), oncogenic signaling (including RAF and BRAF), and the tumor microenvironment (including PDGFR, FGFR, and KIT), thereby inhibiting tumor growth, vascularization, and disease progression
Oxaliplatin	Platinum-based chemotherapeutic agent that induces DNA crosslinks, leading to DNA damage and tumor cell death
Irinotecan	Topoisomerase I inhibitor that interferes with DNA replication and transcription, resulting in apoptosis of rapidly proliferating cancer cells
CB-1158 (INCB001158)	First-in-class selective arginase-1 (ARG1) inhibitor
OATD-02	Second-generation dual arginase 1/arginase 2 (ARG1/ARG2) inhibitor
AZD3965	Selective MCT1 inhibitor

## Data Availability

No new data were created or analyzed in this study. Data sharing is not applicable to this article.

## Abbreviations

The following abbreviations are used in this manuscript:

A2AR	Adenosine A2A Receptor
A2BR	Adenosine A2B Receptor
ACAT2	Acetyl-CoA Acetyltransferase 2
ACTH	Adrenocorticotropic Hormone
AGEs	Advanced Glycation End Products
AgRP	Agouti-Related Peptide
AhR	Aryl Hydrocarbon Receptor
AMPK	AMP-Activated Protein Kinase
AMP	Adenosine Monophosphate
AMT	Amino Acid Metabolism and Transport*
APC	Antigen-Presenting Cell
ARG1	Arginase-1
ARG2	Arginase-2
AVP	Arginine Vasopressin
bHLH	Basic Helix–Loop–Helix
BMAL1	Brain and Muscle ARNT-Like Protein 1
CAF	Cancer-Associated Fibroblast
cAMP	Cyclic Adenosine Monophosphate
CD8	Cluster of Differentiation 8
CD36	Cluster of Differentiation 36
CD39	Ectonucleoside Triphosphate Diphosphohydrolase-1 (ENTPD1)
CD73	Ecto-5′-Nucleotidase (NT5E)
CLOCK	Circadian Locomotor Output Cycles Kaput
CNCDs	Chronic Non-Communicable Diseases
COX-2	Cyclooxygenase-2
CRH	Corticotropin-Releasing Hormone
CRY	Cryptochrome
CTL	Cytotoxic T Lymphocyte
DC	Dendritic Cell
ECM	Extracellular Matrix
ENK	Enkephalin
ER	Estrogen Receptor
ETC	Electron Transport Chain
EV	Extracellular Vesicle
EZH2	Enhancer of Zeste Homolog 2
FABP	Fatty Acid-Binding Protein
FAO	Fatty Acid Oxidation
FATP2	Fatty Acid Transport Protein 2 (SLC27A2)
FBP1	Fructose-1,6-Bisphosphatase 1
FFA	Free Fatty Acid
GABA	Gamma-Aminobutyric Acid
GAPDH	Glyceraldehyde-3-Phosphate Dehydrogenase
GCN2	General Control Nonderepressible 2
G-CSF	Granulocyte Colony-Stimulating Factor
GHT	Geniculohypothalamic Tract
GH	Growth Hormone
GH–IGF-1 axis	Growth Hormone–Insulin-Like Growth Factor 1 Axis
GLUT1	Glucose Transporter 1
GM-CSF	Granulocyte–Macrophage Colony-Stimulating Factor
GRP	Gastrin-Releasing Peptide
Gs	Stimulatory G Protein
H3K18la	Histone H3 Lysine 18 Lactylation
H3K27me3	Trimethylation of Histone H3 Lysine 27
HCC	Hepatocellular Carcinoma
HIF	Hypoxia-Inducible Factor
HIF-1α	Hypoxia-Inducible Factor 1 Alpha
HK	Hexokinase
HK2	Hexokinase 2
HPA	Hypothalamus–Pituitary–Adrenal Axis
IDO	Indoleamine 2,3-Dioxygenase
IDO1	Indoleamine 2,3-Dioxygenase 1
IFN-I	Type I Interferon
IFN-γ	Interferon Gamma
IGF-1	Insulin-Like Growth Factor 1
IGL	Intergeniculate Leaflet
IL-2	Interleukin-2
IL-10	Interleukin-10
IL-12	Interleukin-12
IS	Immune System
JMJDs	Jumonji Domain-Containing Histone Demethylases
Kla	Lysine Lactylation
L-2-HG	L-2-Hydroxyglutarate
LDH	Lactate Dehydrogenase
LDHA	Lactate Dehydrogenase A
LNCaP	Lymph Node Carcinoma of the Prostate (human prostate cancer cell line)
M1	Classically Activated Macrophage
M2	Alternatively Activated Macrophage
MCT1	Monocarboxylate Transporter 1
MDSC	Myeloid-Derived Suppressor Cell
miRNA	MicroRNA
MOESIN	Membrane-Organizing Extension Spike Protein
MR	Magnetic Resonance
mTOR	Mammalian Target of Rapamycin
NAD^+^	Nicotinamide Adenine Dinucleotide (oxidized form)
NADH	Nicotinamide Adenine Dinucleotide (reduced form)
NAMPT	Nicotinamide Phosphoribosyltransferase
NK	Natural Killer Cell
NPAS2	Neuronal PAS Domain Protein 2
NPY	Neuropeptide Y
NSCLC	Non-Small-Cell Lung Cancer
OS	Overall Survival
OXPHOS	Oxidative Phosphorylation
PACAP	Pituitary Adenylate Cyclase-Activating Polypeptide
PD-1	Programmed Cell Death Protein 1
PDAC	Pancreatic Ductal Adenocarcinoma
PDH	Pyruvate Dehydrogenase
PDK1	Pyruvate Dehydrogenase Kinase 1
PDK3	Pyruvate Dehydrogenase Kinase 3
PD-L1	Programmed Death-Ligand 1
PER	Period Circadian Regulator Protein
PFS	Progression-Free Survival
PHDs	Prolyl Hydroxylases
PKA	Protein Kinase A
PKM2	Pyruvate Kinase M2
PR	Progesterone Receptor
PGE2	Prostaglandin E2
RHT	Retinohypothalamic Tract
ROS	Reactive Oxygen Species
SAM	Sympatho–Adrenomedullary Axis
SCN	Suprachiasmatic Nucleus
SOD2	Superoxide Dismutase 2
SREBP	Sterol Regulatory Element-Binding Protein
STING	Stimulator of Interferon Genes
T2DM	Type 2 Diabetes Mellitus
TAM	Tumor-Associated Macrophage
TAN	Tumor-Associated Neutrophil
TCR	T-Cell Receptor
TDO	Tryptophan 2,3-Dioxygenase
TGF-β	Transforming Growth Factor Beta
TIL	Tumor-Infiltrating Lymphocyte
TLA	Three-Letter Acronym
TLR8	Toll-Like Receptor 8
TME	Tumor Microenvironment
TNBC	Triple-Negative Breast Cancer
TNF	Tumor Necrosis Factor
TNF-α	Tumor Necrosis Factor Alpha (α)
TP53	Tumor Protein p53
Treg	Regulatory T Cell
VEGF	Vascular Endothelial Growth Factor
VEGFA	Vascular Endothelial Growth Factor A
VIP	Vasoactive Intestinal Peptide
